# Identification and Characterization of NF-Y Transcription Factor Families in the Monocot Model Plant *Brachypodium distachyon*

**DOI:** 10.1371/journal.pone.0021805

**Published:** 2011-06-30

**Authors:** Shuanghe Cao, Roderick W. Kumimoto, Chamindika L. Siriwardana, Jan R. Risinger, Ben F. Holt

**Affiliations:** Department of Botany and Microbiology, University of Oklahoma, Norman, Oklahoma, United States of America; East Carolina University, United States of America

## Abstract

**Background:**

Nuclear Factor Y (NF-Y) is a heterotrimeric transcription factor composed of NF-YA, NF-YB and NF-YC proteins. Using the dicot plant model system *Arabidopsis thaliana* (Arabidopsis), NF-Y were previously shown to control a variety of agronomically important traits, including drought tolerance, flowering time, and seed development. The aim of the current research was to identify and characterize NF-Y families in the emerging monocot model plant *Brachypodium distachyon* (Brachypodium) with the long term goal of assisting in the translation of known dicot NF-Y functions to the grasses.

**Methodology/Principal Findings:**

We identified, annotated, and further characterized 7 NF-YA, 17 NF-YB, and 12 NF-YC proteins in Brachypodium (BdNF-Y). By examining phylogenetic relationships, orthology predictions, and tissue-specific expression patterns for all 36 BdNF-Y, we proposed numerous examples of likely functional conservation between dicots and monocots. To test one of these orthology predictions, we demonstrated that a BdNF-YB with predicted orthology to Arabidopsis floral-promoting NF-Y proteins can rescue a late flowering Arabidopsis mutant.

**Conclusions/Significance:**

The Brachypodium genome encodes a similar complement of NF-Y to other sequenced angiosperms. Information regarding NF-Y phylogenetic relationships, predicted orthologies, and expression patterns can facilitate their study in the grasses. The current data serves as an entry point for translating many NF-Y functions from dicots to the genetically tractable monocot model system Brachypodium. In turn, studies of NF-Y function in Brachypodium promise to be more readily translatable to the agriculturally important grasses.

## Introduction

NUCLEAR FACTOR Y (NF-Y, also called heme-activated protein (HAP) or CCAAT binding factor (CBF)) is a heterotrimeric transcription factor that is found in all sequenced eukaryotes. The transcriptional activation of hundreds to possibly thousands of mammalian genes is regulated by NF-Y binding to *cis*-elements containing the highly conserved core sequence *CCAAT* [1], [2]. Although there is considerable disparity in these estimates, recent reports suggest that ∼7–8% of human promoters have functional *CCAAT* motifs [1], [3]. NF-Y bound promoters appear to be fairly evenly distributed between housekeeping and tissue/developmental specific genes. Additionally, many cell cycle genes require NF-Y for proper timing of expression [4]. Not surprisingly, various mutations in both mouse and Drosophila NF-Y are lethal [5], [6]. Due to the variety of NF-Y regulated targets and its importance during development, much attention has been directed towards genetically and biochemically dissecting the ubiquitous roles of NF-Y as transcriptional activators [7], [8], [9].

In mammals, the functional NF-Y transcription factor is composed of three unique subunits, each of which is encoded by a single gene [8], [9], [10]. These subunits, called NF-YA, NF-YB, and NF-YC, assemble sequentially to form the functional NF-Y transcription factor [11]. NF-YB and NF-YC initially form a dimer in the cytoplasm and then translocate to the nucleus where they interact with NF-YA and bind *CCAAT* sites in target promoters [4], [12]. Only the heterotrimeric complex is thought to bind *CCAAT* sites. NF-YB and NF-YC are related to H2B and H2A histones, respectively, while NF-YA appears to be a unique protein [9], [13], [14]. While there have been many publications regarding the functions, biochemistry, and transcriptional targets for NF-Y in yeast and mammals, our collective understanding of NF-Y roles in the plant lineage lags further behind.

The paucity of NF-Y data for the plant lineage is at least partly due to overlapping functionality between individual family members; instead of single genes, plants have gene families encoding each NF-Y subunit type. For example, the *Arabidopsis thaliana* (Arabidopsis) and *Triticum aestivum* (wheat) genomes encode at least 36 and 37 NF-Y subunits, respectively (>10 unique genes for each family [15], [16]). Because the mature NF-Y transcription factor is composed of one subunit from each family, Arabidopsis can theoretically produce 1,690 unique NF-Y complexes (10 NF-YA X 13 NF-YB X 13 NF-YC = 1,690 [15]; note that this does not include splice variants). Unique expression patterns (tissue, developmental, stress responsive, etc.) for each gene should considerably reduce this theoretical value, but defining the roles of specific genes remains complicated. Because of this large amount of combinatorial diversity, there is great potential to utilize engineered NF-Y to subtly modify gene expression outputs [15].

Although no complete NF-Y complex for a given process or gene regulatory target has been demonstrated in the plant lineage, individual NF-Y subunits are known to be required for a growing number of important plant processes: NF-Y have roles in embryogenesis [17], [18], blue light signaling [19], drought resistance (and concomitant crop productivity) [20], [21], photoperiod-dependent flowering [22], [23], [24], [25], [26], [27], endoplasmic reticulum (ER) stress [28], abscisic acid (ABA) perception [19], [29], nitrogen-fixing nodule development [30], [31], [32], photosynthesis [33], [34], and root elongation [35]. Research on NF-Y roles in flowering time and drought resistance is particularly interesting as modifications in those phenotypes hold considerable promise for improving both food and biofuel crops [36].

Relative to NF-Y roles in flowering, there is already direct evidence for overlapping functionality between family members. As identified by various reverse genetic approaches, at least two *NF-YB* (*NF-YB2* and *3*) and three *NF-YC* (*NF-YC3*, *4*, and *9*) are involved in photoperiod-dependent flowering [22], [26]. Because single loss of function alleles have only moderate [22], [23], [24] or no [26] significant affect on flowering time, these genes would not likely have been identified by traditional forward genetic approaches. Reverse genetic approaches targeted at functional gene discovery are facilitated by characterization and annotation of gene families. Additionally, predictions of shared protein functions between species are improved by phylogenetic trees [37] and detailed knowledge about expression patterns [38]. In fact, evidence for the overlapping roles of *NF-YC3*, *4*, and *9* in flowering was originally deduced from their shared developmental- and tissue-specific co-expression patterns with the known floral regulator *CONSTANS* (*CO* [39]). Additionally, NF-Y expression analyses in wheat [16] led to the identification of NF-Y subunits with specific roles in photosynthesis [33], [40]. Therefore, to provide this type of detailed information, several groups have recently developed phylogenetic trees, annotations, and various types of family-wide *NF-Y* expression analyses [15], [16], [41], [42], [43]. To date, essentially complete characterizations/annotations in the plant lineage are available for the Arabidopsis and *Oryza sativa* (rice) NF-Y families [15], [43]. However, relative to important temperate grasses and herbaceous energy crops, only partial data is available for wheat [16] and NF-Y families have not been described for their emerging monocot model plant Brachypodium.

Due to a number of favorable features, including its small stature, simple growth conditions, rapid life cycle, and genetic tractability, Brachypodium is a very attractive model system for the monocot lineage [44], [45]. Brachypodium is a member of the subfamily Pooideae and is closely related to wheat, oats, and barley [46]. In addition to its obvious utility as a model for the world's most important food crops, Brachypodium is also a highly tractable model for emerging biofuel crops, such as switchgrass and *Miscanthus* [47]. In 2010, a draft sequence of the complete Brachypodium genome sequence (diploid inbred line Bd21) was released [48]. This information is publicly accessible (www.brachypodium.org) and is particularly useful for exploring gene families and predicting functional conservation between species.

The aim of the current research was to facilitate further investigations of NF-Y transcription factors in the grasses. In this paper we characterize the three unique “Bd” NF-Y families in Brachypodium, including their phylogenetic relationships, expression analyses for several tissue types, and a proposed nomenclature for each family/gene. As in Arabidopsis, we find that Brachypodium has ∼36 *BdNF-Y* genes with highly variable, tissue-specific expression patterns. Additionally, we examine orthology between NF-Y in Brachypodium and Arabidopsis and demonstrate the utility of these functional genomic comparisons with a flowering time rescue assay. This research serves as an entrée to the NF-Y in an emerging monocot model system where rapid progress on the roles of NF-Y in development and stress responses is expected.

## Results and Discussion

### Discovery and annotation of the Brachypodium BdNF-Y

Because additional, broadly applicable concepts are further developed in previous Arabidopsis, wheat, and rice NF-Y papers, they are recommended as complementary reading to the current Brachypodium manuscript [15], [16], [41], [42], [43]. We and others previously characterized the NF-Y families in Arabidopsis and found 10 *NF-YA*, 13 *NF-YB*, and 13 *NF-YC* encoding genes [15], [41], [42]. Full length and conserved regions from each of the 36 predicted Arabidopsis NF-Y were used here to sequentially BLAST [49] search the Brachypodium database (www.brachypodium.org). We also found 36 total NF-Y proteins in Brachypodium, although the distributions within families were different from Arabidopsis - we identified 7 NF-YA, 17 NF-YB, and 12 NF-YC proteins (Table 1). Based on recent monocot transcription factor nomenclature suggestions [50], each protein was named with a two-letter code corresponding to *Brachypodium distachyon* (Bd), followed by NF-Y and the family designation (NF-YA, B, or C), and finally a number. Because there are no existing functional descriptions or published loss of function alleles for the BdNF-Y, there are no pre-existing number schemes for these families. Additionally, phylogenetic analyses (below) suggested that there are no easy protein-for-protein relationships to simplify the numbering scheme between lineages. Therefore, we started the numbering at one for each family and continued in steps of one according to their chromosomal positions. For example, BdNF-YA1 (Bradi1g11800) is nearest the top of chromosome one and BdNF-YA7 (Bd4g01820), the last A family member, is on chromosome four. We note that we found no BdNF-YA or C subunits on chromosome five, and no BdNF-YB on chromosomes four and five.

**Table 1:** Annotation of the *Brachypodium distachyon* NF-Y families.

Brachy NF-YA Family			Brachy NF-YB Family			Brachy NF-YC Family		
Name	IBI	Putative *At* Orthologs	Name	IBI	Putative *At* Orthologs	Name	IBI	Putative *At* Orthologs
BdNF-YA1	Bradi1g11800	NF-YA9, 4	BdNF-YB1	Bradi1g21900	NF-YB3, 2	BdNF-YC1	Bradi1g01300	NF-YC10
BdNF-YA2	Bradi1g13680	NF-YA5, 6	BdNF-YB2	Bradi1g43460	NF-YB6, 9	BdNF-YC2	Bradi1g32200	NF-YC2, 1, 3, 4, 9
BdNF-YA3	Bradi1g21760	NF-YA5, 8	BdNF-YB3	Bradi1g43470	NF-YB1, 3	BdNF-YC3	Bradi1g67980	NF-YC4, 1
BdNF-YA4	Bradi1g72960	NF-YA5, 8	BdNF-YB4	Bradi1g43480	NF-YB6, 9	BdNF-YC4	Bradi2g21290	NF-YC11
BdNF-YA5	Bradi3g57320	NF-YA5, 8	BdNF-YB5	Bradi1g43490	*NSH*	BdNF-YC5	Bradi3g05270	NF-YC9, 3
BdNF-YA6	Bradi4g01380	NF-YA5, 6	BdNF-YB6	Bradi1g60030	NF-YB3, 2	BdNF-YC6	Bradi3g17790	NF-YC2, 9, 3
BdNF-YA7	Bradi4g01820	NF-YA5, 7	BdNF-YB7	Bradi2g15800	NF-YB8, 10	BdNF-YC7	Bradi3g17800	NF-YC2, 4, 1
			BdNF-YB8	Bradi2g22940	NF-YB8, 3, 10	BdNF-YC8	Bradi3g17810	NF-YC2, 4, 1
			BdNF-YB9	Bradi2g54200	NF-YB10, 8	BdNF-YC9	Bradi3g17820	NF-YC2, 9, 3
			BdNF-YB10	Bradi2g60030	NF-YB4, 5	BdNF-YC10	Bradi3g39280	NF-YC9, 3
			BdNF-YB11	Bradi2g60050	NF-YB5, 2	BdNF-YC11	Bradi4g16840	NF-YC11
			BdNF-YB12	Bradi3g15670	NF-YB3, 2	BdNF-YC12	Bradi4g33290	NF-YC9, 3
			BdNF-YB13	Bradi3g17350	NF-YB1, 8			
			BdNF-YB14	Bradi3g17360	NF-YB1, 8			
			BdNF-YB15	Bradi3g17990	NF-YB1, 3			
			BdNF-YB16	Bradi3g34930	NF-YB13, 12			
			BdNF-YB17	Bradi3g56400	NF-YB6, 9			

Names for each family are ordered based on chromosomal position - i.e., lowest numbers start at the top of chromosome 1 and continue to the bottom of chromosome 4 for each family (there are no predicted BdNF-Y on chromosome 5). Each BdNF-Y is associated with a single International Brachypodium Initiative (IBI) number and putative Arabidopsis (At) orthologs. Orthologs were generated by Blast searches with the conserved domains from each protein, as implemented by InParanoid7 (http://inparanoid.sbc.su.se/cgi-bin/index.cgi) [66]. Using the default E-value cutoff (<0.01), the top two scoring Arabidopsis orthologs are listed for each BdNF-Y protein. Where more than two orthologs are listed, multiple Arabidopsis proteins had identical orthology scores; “*NSH*”  =  no significant hits. Underlined Arabidopsis orthologs were highly significant matches to Brachypodium at <1e-20.

### Multiple Alignments and Phylogenetic Analyses of BdNF-Y

**Figure 1:**
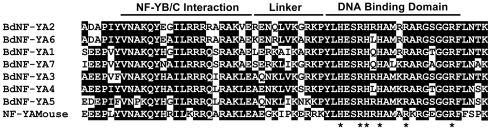
BdNF-YA family multiple alignment. Multiple alignments were performed by ClustalX [51] as implemented by Molecular Evolutionary Genetics Analysis (MEGA) software, version 4.0 [52]. Amino acid sequences corresponding to the conserved regions for all BdNF-YA and the *Mus musculus* NF-YA (NF-YAMouse) are shown. NF-YAMouse is included as an outgroup to root the phylogenetic trees (below) and for comparison. Amino acids in black boxes/white letters are identical in at least 50% of all aligned sequences. Asterisks below H and R residues represent amino acids known to be specifically required for DNA binding [53]. Regions identified as required for DNA binding or subunit interactions for NF-YA, as well as the NF-YB and NF-YC subunits in Figures 2 and 3 below, were previously defined in the yeast and mammalian literature [10], [11], [53], [54], [55], [56], [57], [89].

**Figure 2:**
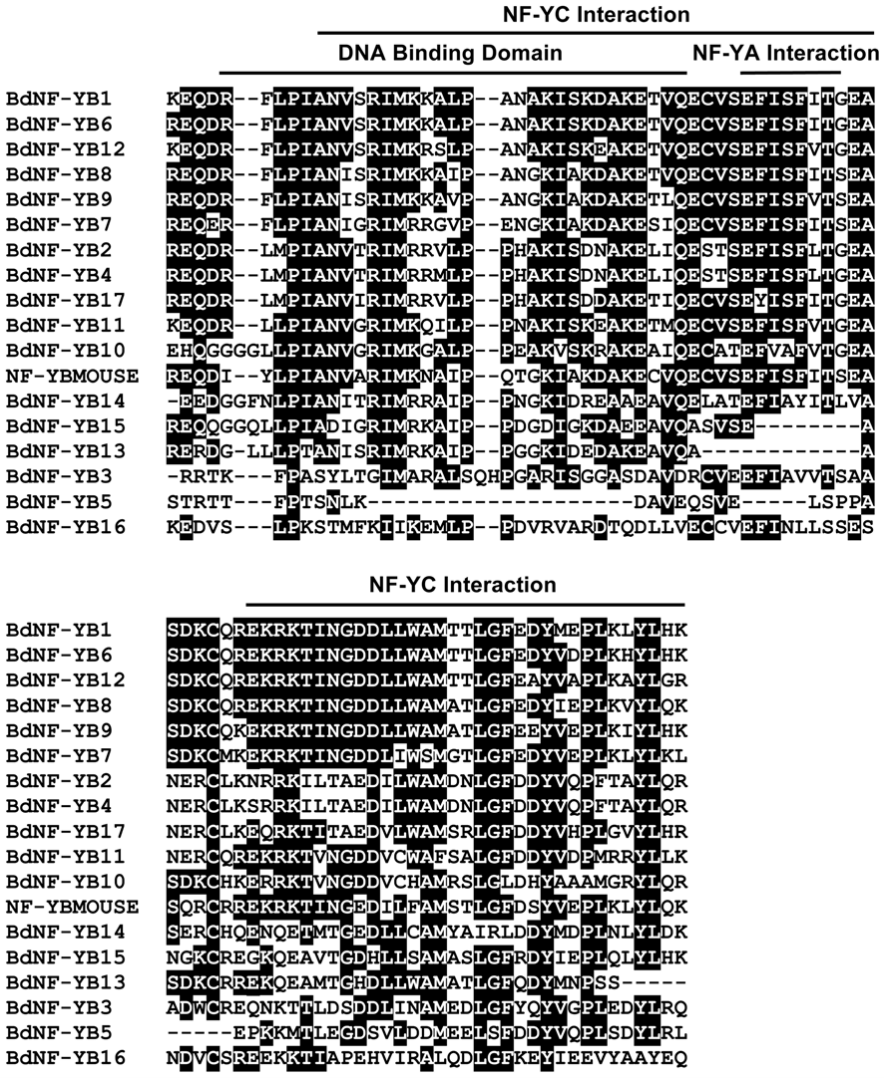
BdNF-YB family multiple alignment. Constructed as in Figure 1.

**Figure 3:**
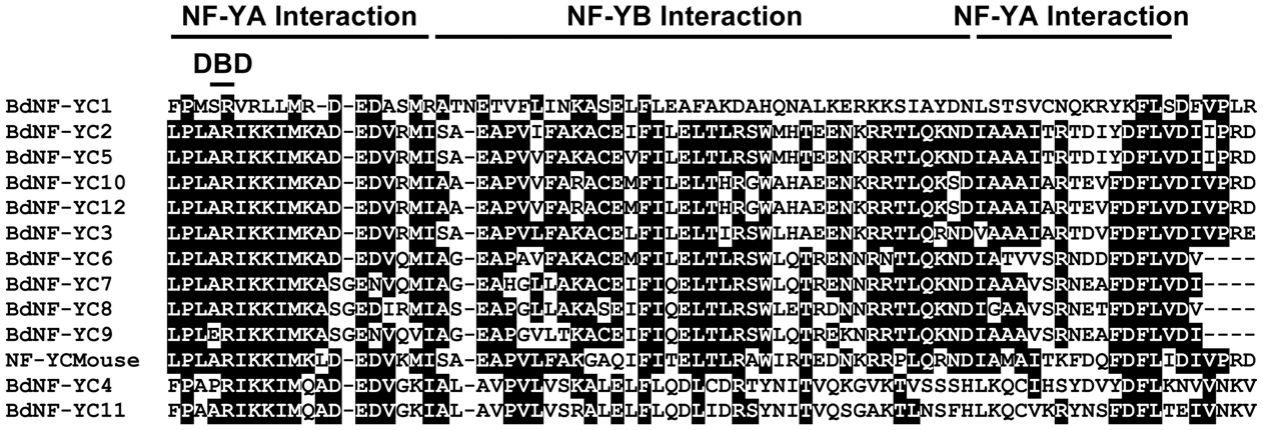
BdNF-YC family multiple alignment. Constructed as in Figure 1.

For each BdNF-Y family, multiple alignments were developed using ClustalX [51] as implemented in MEGA 4.0 [52]. As with Arabidopsis NF-Y, BdNF-Y proteins have clear regions of homology flanked by largely non-conserved sequences [15]. In mammals and yeast, the conserved regions are known to be necessary and sufficient for heterodimerization, heterotrimerization, and DNA interactions at *CCAAT* sites [11], [53], [54], [55], [56], [57]. The multiple alignments in Figures 1, 2, 3 show the conserved regions for each BdNF-Y family with the interaction domains highlighted (inferred from mammals or yeast; Fasta files for both the conserved regions and full length BdNF-Y are in Dataset S1). In mammals and yeast, NF-YA and NF-YC subunits are additionally flanked by numerous glutamines and hydrophobic residues and these regions are known to be involved in transcriptional activation [9], [58], [59] (see alignments of entire protein sequences, Figures S1, S2, S3). There is conservation of this theme in Brachypodium and throughout the plant lineage [15], but these sequences are quite variable, even between members of the same NF-Y family in the same species, and will need to be tested individually for transcriptional activation ability.

**Figure 4:**
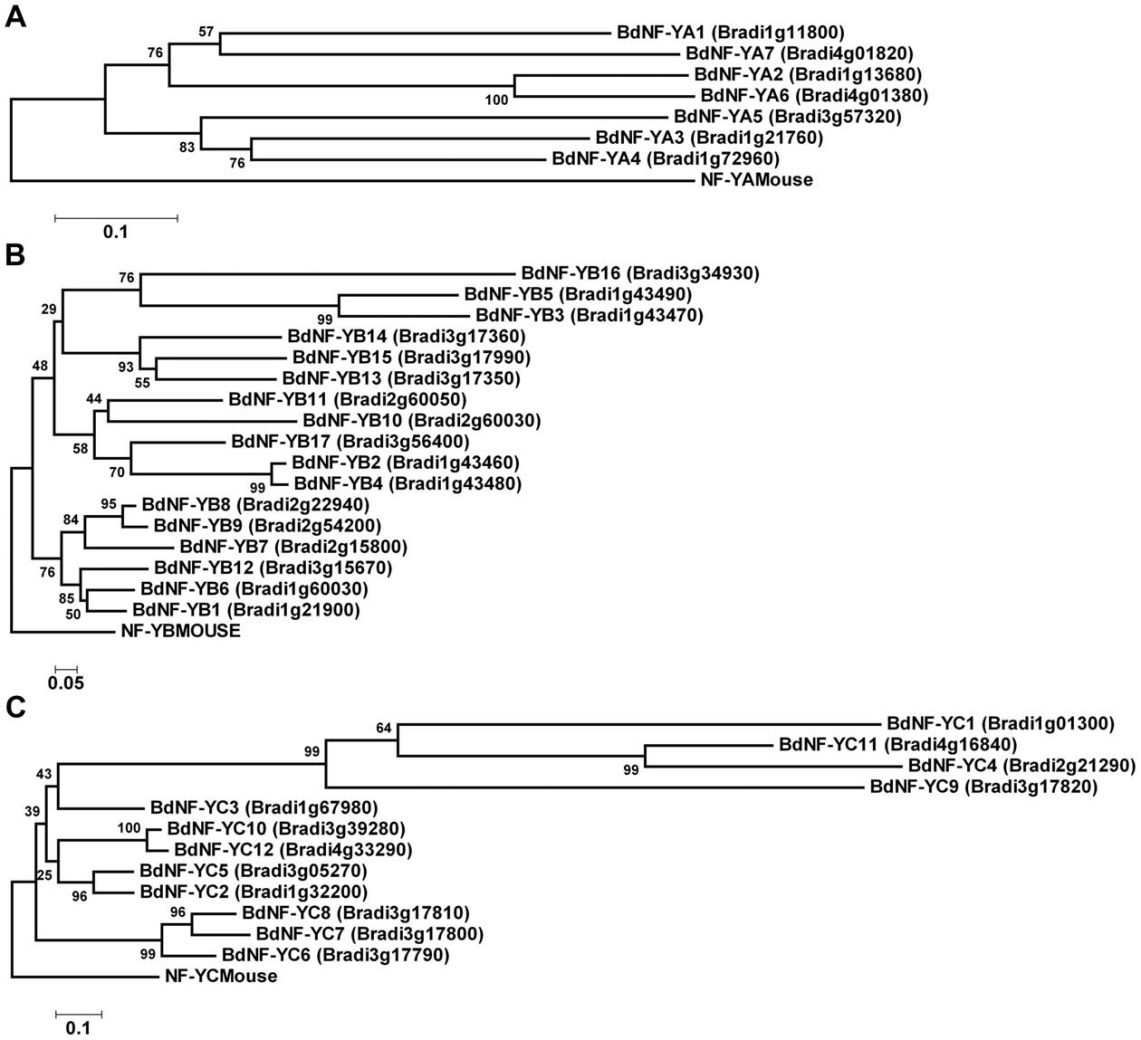
Phylogenetic trees for the Brachypodium BdNF-Y families. **A**) BdNF-YA phylogenetic tree. **B**) BdNF-YB phylogenetic tree. **C**) BdNF-YC phylogenetic tree. For each BdNF-Y family, phylogenetic trees were constructed by neighbor-joining as implemented by Molecular Evolutionary Genetics Analysis (MEGA) software, version 4.0 [52]. Reliability values at each branch represent bootstrap samples (10,000 replicates). Note that BdNF-YB16 is orthologous to the DR1-related proteins and these are sometimes shown as a separate, NF-YB-like family [11], [16], [90]. There is no functional data to suggest that either choice is “correct,” but they are more closely related to NF-YB proteins than any other best match in BLAST searches. Thus, they are included here as related, but divergent NF-YB family members.

The multiple alignments for each BdNF-Y family were used to develop neighbor-joining phylogenetic trees (Figure 4A–C). One immediately apparent observation was the overall shape similarity between previously generated Arabidopsis NF-Y phylogenetic trees [15] and those independently developed here for Brachypodium. In both species, the NF-YA subunits are generally found on the tree as a series of paralogs (although Brachypodium has three fewer NF-YA than Arabidopsis) that are all roughly equidistant from each other and the mouse ortholog. This is in contrast to the BdNF-YB and BdNF-YC where there are clear blocks of proteins that are more closely related to the respective mouse orthologs and additional blocks that are considerably more divergent (Figure 4B–C). Differences in post-duplication evolutionary patterns between NF-Y families were previously identified for both Arabidopsis and rice [60], where the authors concluded that only the NF-YB and NF-YC duplicates were undergoing asymmetric evolution. Asymmetric evolution of duplicates may indicate that the NF-YB and NF-YC families experienced relaxed selective constraints to change following their respective duplication events.

Structural and functional data suggests that NF-YA proteins might be more evolutionarily constrained than either NF-YB or NF-YC proteins. For example, crystal structures of the mammalian NF-YB/NF-YC dimer, and modeled interactions between the entire NF-Y complex and DNA, predict that NF-YA subunits are specifically responsible for all physical contacts with the *CCAAT* sequence [57]. Mutations in this core DNA pentamer result in complete or near complete loss of NF-Y binding [2], [61]. For mammalian NF-YA, three histidine and three arginine residues are absolutely required for DNA binding [53] and these residues are conserved in all seven BdNF-YA (asterisks in Figure 1). These same residues are conserved in 10/10 Arabidopsis, 9/10 rice, and 10/10 wheat NF-YA [15], [16], [43]. Therefore, this specific function is highly conserved in essentially all NF-YA and likely acts as a strong constraint against post duplication evolutionary divergence.

Alternatively, the NF-YB and NF-YC subunits are thought to make DNA contact with the flanking regions surrounding the *CCAAT* pentamer [57] and might be under weaker constraints to change (including *CCAAT*, NF-Y binding sites are approximately 13bp in length [61]). Supporting this idea, the flanking regions of NF-Y binding sites are much more loosely conserved by comparison to the central *CCAAT* sequence [61]. This data might also explain why positional weight matrices based on mammalian data do not find concentrations of NF-Y binding sites at expected locations in plant promoters [15] - i.e., both the regions surrounding *CCAAT* pentamers in DNA and the NF-YB/NF-YC subunits responsible for these interactions are rapidly diversifying in the plant lineage. Testing this hypothesis will require the discovery of biologically relevant NF-YA/B/C combinations in the plant lineage combined with approaches such as chromatin immunoprecipitation and sequencing [1]. This information will likely provide important insights when considering the use of NF-Y as regulators of synthetic gene constructs [62], [63] or for subtly modifying their functions in largely natural settings.

### Comparative phylogenetic trees and orthology predictions

The primary aim of this research was to provide comparative functional insights between Arabidopsis, where there is growing evidence for the roles of numerous NF-Y, and Brachypodium, where no publications regarding NF-Y function have been published. Orthology predictions are important for transferring functional information between species [64] and results from Brachypodium are more likely to be directly translatable to agriculturally important monocots, particularly the temperate grasses such as wheat and barley [65]. Using the conserved regions from each Brachypodium and Arabidopsis [15] NF-Y family (Dataset S1, S2), we developed neighbor-joining phylogenetic trees for visual analysis of potential relationships (Figures S4, S5, S6). For ease of visualizing functional relationships, Figures S4, S5, S6 were additionally annotated with the known Arabidopsis NF-Y functions. Although these trees were helpful to establish a general feel for ancestry and distance between individual NF-Y, many of the branches were poorly supported by bootstrap analyses.

To strengthen the arguments for likely functional conservation between specific Arabidopsis and Brachypodium NF-Y, we additionally searched for Arabidopsis orthologs for each BdNF-Y using InParanoid7 [66], [67]. Instead of inferring orthology directly from phylogenetic trees, InParanoid7 uses BLAST [49] to make protein alignments and then applies a stringent filtering algorithm to avoid erroneous fragment matches [66]. Using their web-based BLAST portal, each BdNF-Y was surveyed and the top two Arabidopsis orthology matches were recorded (Table 1, “Putative At Orthologs”). We additionally highlighted orthology matches with particularly strong e-value scores (<1e-20, underlined orthologs in Table 1). Further, orthology data comparing Brachypodium to rice and wheat is available as supplemental data (Table S1; Note that we find orthology matches to numerous wheat TaNF-Y that were not annotated in a previous publication [16]. This is likely due to the incomplete draft nature of that genome sequence).

BdNF-YA have highly significant (<1e-20) orthology matches to NF-YA5, 8, and 9. The best orthology match for six out of seven BdNF-YA proteins is to Arabidopsis NF-YA5, and three of those matches are highly significant. Regulation of NF-YA5 has been implicated in drought tolerance, thus BdNF-YA3, 4, and 5 represent the most likely candidates for similar functions in Brachypodium. For the NF-YB and NF-YC families, the orthology matches to Arabidopsis were more evenly distributed (i.e., good matches to numerous NF-YB and NF-YC were found). For example, BdNF-Y2, 4, and 17 have highly significant matches to NF-YB6 (LEC1-Like, L1L) and NF-YB9 (LEAFY COTYLEDON 1, LEC1). LEC1 and L1L are important during Arabidopsis embryogenesis and constitute a unique subclass of NF-YB proteins [17], [68], [69]. For the known Arabidopsis regulators of photoperiod-dependent flowering time, NF-YB2 and NF-YB3, Brachypodium has highly significant orthology matches to BdNF-YB1, 6, and 12. Arabidopsis NF-YB2 and NF-YB3 are closely related proteins that appear to have arisen from a relatively recent duplication event [15] and this appears to be true for BdNF-YB1 and BdNF-YB6 as well (Figure 4B). Based on the phylogenetic and orthology data presented here, similar comparative analyses can be made for any BdNF-Y, thus providing a starting point for hypothesis generation and functional analyses.

### Tissue specific BdNF-Y expression patterns

While BdNF-Y annotations, phylogenies, and orthology predictions can provide a useful starting point for BdNF-Y investigations, gene expression data could improve the quality of these predictions and reduce the numbers of starting options for time-consuming functional tests. For example, the above orthology analyses highlighted three likely BdNF-YB candidates for functional similarity to the Arabidopsis flowering time regulators NF-YB2 and NF-YB3. During the initiation of photoperiod-dependent flowering, NF-YB2 and NF-YB3 are thought to physically interact (primarily through NF-YC interactions) with CO in the vascular system of ∼one to two week old leaves [22], [26], [70]. Additionally, both Arabidopsis and Brachypodium initiate flowering at similar times after germination under appropriate photoperiods [71]. Thus, a reasonable hypothesis is that the appropriate BdNF-YB(s) for this response will have highly significant orthology scores to NF-YB2 and NF-YB3 and also be expressed in one to two week old shoots.

To generate a starting expression dataset, we developed primer sets for each *BdNF-Y* suitable for quantitative reverse transcriptase polymerase chain reactions (qRT-PCR, Table S2). So that direct comparisons of expression could be made between different *BdNF-Y*, optimal primer pairs with approximately equal PCR efficiencies for both genomic DNA and experimental cDNA were developed for each gene (difference <0.04, see Materials and Methods). For each plate, data was then calibrated using standard curves based on genomic DNA amplification and then further normalized with a reference gene (*UBC18* [72]). We then examined gene expression by qRT-PCR in roots and shoots (both 14 days old), inflorescences, and caryopses for all 36 *BdNF-Y*. Three biological replicates were examined for each *BdNF-Y* and tissue type.

#### NF-YA expression

**Figure 5:**
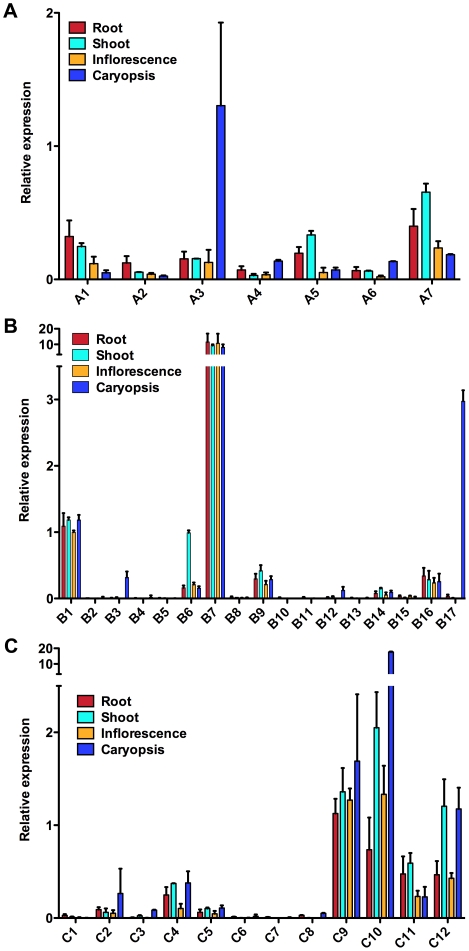
*BdNF-Y* expression analyses by qRT-PCR. **A**) Expression of *BdNF-YA* genes. Note that the qPCR data from all primer sets are calibrated with their standard curves from genomic DNA so that expression can be compared for different tissues for a single *BdNF-Y* and between different *BdNF-Y* (see Methods). **B**) Expression of *BdNF-YB* genes. Note that a segmented Y-axis and bars are used to accommodate the uniformly high expression level of *BdNF-YB7*. **C**) Expression of *BdNF-YC* genes. As in 5B, a segmented Y-axis and bar is used to accommodate high expression in *BdNF-YC10*, specifically in the caryopses.

By comparison to the *BdNF-YB* and *BdNF-YC* families, all of the *BdNF-YA* are expressed, but at generally lower levels (Figure 5A). *BdBF-YA3* expression in caryopses is a notable exception as it is expressed ∼7 fold higher than any other BdNF-YA in caryopses and 2-fold higher than the next highest expressed A subunit (*BdNF-YA7* in shoots). Because NF-YA have known roles in embryogenesis, likely mediated through signaling interactions with the seed dormancy-promoting hormone ABA [19], [21], [29], [73], [74], [75], BdNF-YA3 is an interesting target for future studies of seed development and somatic embryogenesis. Currently much more is known about the specific functions of the B and C subunits, but it is not surprising to find that *BdNF-YA* are generally expressed at lower levels. In Arabidopsis the accumulation of most *NF-YA* transcripts is regulated by the micro RNA miR169 [76]. Downregulation of miR169 can lead to NF-YA upregulation under very specific environmental conditions, such as drought or nitrogen starvation [21], [77]. From our own analyses, we know that most overexpressed *NF-YA* cause extreme dwarfism and low seed set in Arabidopsis, although these lines accumulate very little transgenic protein (C. Siriwardana and B. Holt, unpublished). Based on the conservation of miR169 in Brachypodium [78], it is likely that similar mechanisms control the accumulation of *BdNF-YA* mRNA and protein.

#### NF-YB expression

Expression of the *BdNF-YB* genes was quite variable and *BdNF-YB7*, in particular, was very strongly expressed in all tissue types (>10 fold higher than most other B subunits, Figure 5B). *BdNF-YB1* has a similarly uniform expression pattern, but is expressed at a relatively lower level than *BdNF-YB7* (∼10 fold less). BdNF-YB7 is orthologous to NF-YB8 and 10 and neither of those genes appear to be particularly strongly expressed in Arabidopsis (with the exception of NF-YB10 in root tips, [15]). Thus it is interesting to speculate that this uniformly high level of *BdNF-YB7* expression in the monocot lineage might represent a unique function, such as the expression of a housekeeping gene. Alternatively, mammals also have uniformly strong expression of their single *NF-Y* genes in essentially all cell types, yet mammalian NF-Y complexes regulate everything from highly conditionally specific expression to ubiquitously expressed genes [1], [61].

As previously mentioned, BdNF-YB1, 6, and 12 are orthologous to the flowering time regulators NF-YB2 and NF-YB3. From the expression analysis, only *BdNF-YB1* and *BdNF-YB6* appear to have any expression in 14 day-old shoots. Based on this expression analysis and the phylogenetic data discussed above, BdNF-YB1 and BdNF-YB6 are obvious targets for future investigations on photoperiod-dependent flowering in Brachypodium (the value of this prediction for BdNF-YB6 is demonstrated below).

The best LEC1 (NF-YB9) and L1L (NF-YB6) orthology matches were to BdNF-YB2, 4, and 17. In Arabidopsis, LEC1 and L1L are the only members of a subclass of NF-YB proteins. This classification is based on the presence of 16 amino acids only shared by LEC1 and L1L [17]. Neither is expressed in vegetative organs and flowers; detectable expression is confined to seeds and developing siliques [17], [18]. As expected for LEC1 orthologs, *BdNF-YB2*, *4*, and *17* are essentially non-expressed in all tissues, with one exception: *BdNF-YB17* is very highly induced in caryopses. BdNF-YB17 also shares the first 14 of the 16 unique LEC1/L1L amino acids [17]. In contrast, BdNF-YB2 and BdNF-YB4 share only 12 of the 16 LEC1/L1L conserved amino acids and the differences are found spread throughout the conserved region. Therefore, BdNF-YB17 appears to be a very good initial target for studies of Brachypodium embryogenesis.

#### NF-YC expression

Similar to the *BdNF-YBs*, *BdNF-YC* expression is highly variable throughout the family (Figure 5C). On chromosome 3 there are four tandemly arranged *BdNF-YC* genes (*6*, *7*, *8*, and *9*). Because these four BdNF-YC are orthologous to NF-YC1, 2, 3, 4 and 9, a likely monophyletic group with overlapping functions in Arabidopsis [15], [41], [42], research on them might be rendered difficult due to their close chromosomal proximities. Potentially reducing this complexity, only BdNF-YC9 appears to be well expressed from this group. Recent evidence suggests that Arabidopsis *NF-YC2* is involved in ER stress, but is non-expressed or expressed at very low levels in the absence of actual stress [15], [28]. Thus, finding BdNF-YC orthologs with similar expression might require the opposite approach to the previous examples - i.e., one might look for low expressed NF-YC2 orthologs (such as BdNF-YC2, 6, 7, and 8) and examine their expression following ER stress induction.

Conversely, shoot expressed members of this NF-YC1, 2, 3, 4, 9 orthology group are good targets for studies of flowering time. However, examination of the Brachypodium and Arabidopsis NF-YC phylogenetic tree (Figure S6) suggests that this analysis is less straightforward than for the BdNF-YBs. BdNF-YC2, 3, and 5 are most closely related to the known Arabidopsis floral promoting NF-YC, but all of these genes are expressed at low levels in Brachypodium shoots. BdNF-YC9, 10, and 12 are expressed at high levels in shoots, but are not as closely related. Previous expression analyses of both the floral promoting NF-YB and NF-YC proteins suggest that they are also the most uniformly and highly expressed NF-Ys throughout the Arabidopsis life cycle [15]. Therefore, in this instance, one must decide whether to place greater value on expression or orthology as a predictor of shared functionality across these distant lineages.

As with the BdNF-YA and BdNF-YC families, there is one *BdNF-YC* with very high expression in caryopses, *BdNF-YC10*. Biochemical and genetic interaction studies to examine the entire set of co-induced BdNF-Y in caryopses, *BdNF-YA3*, *BdNF-YB17*, and *BdNF-YC10*, should illuminate essential roles for NF-Y in both embryogenesis and ABA responses in the monocot lineage. Comparative loss of function analyses, which are becoming increasingly possible in the Brachypodium model [79], will help to define their overlapping and unique roles in these processes.

### BdNF-Y by AtNF-Y orthology predictions have demonstrated predictive value

To test the functional validity of our comparative approach, we cloned *BdNF-YB3* and *BdNF-YB6* and examined whether or not they could alter flowering time in Arabidopsis. We chose BdNF-YB6 because it was predicted to share a relatively recent ancestor with two positive regulators of flowering, NF-YB2 and NF-YB3 (Figure S5 [22]). Further, orthology scores between BdNF-YB6 and NF-YB2/NF-YB3 were very significant (2.8e-42 and 5.6e-43, respectively, Table 1). BdNF-YB3 was chosen as an outlier control because it was phylogenetically distant from NF-YB2 and NF-YB3 (Figure S5). Additionally, the best orthology match for BdNF-YB3 was to NF-YB1 (6.0e-13, Table 1). NF-YB1 overexpression was previously shown to cause *late* flowering, suggesting it can act as a negative regulator of flowering [20], [27]. Based on the phylogenetic and orthology analyses presented here, we hypothesized that overexpression of *BdNF-YB6* would drive early flowering in Col-0 and rescue the late flowering phenotype of *nf-yb2 nf-yb3* plants. Alternatively, *BdNF-YB3* was hypothesized to have either negative or neutral effects on flowering time.

**Figure 6:**
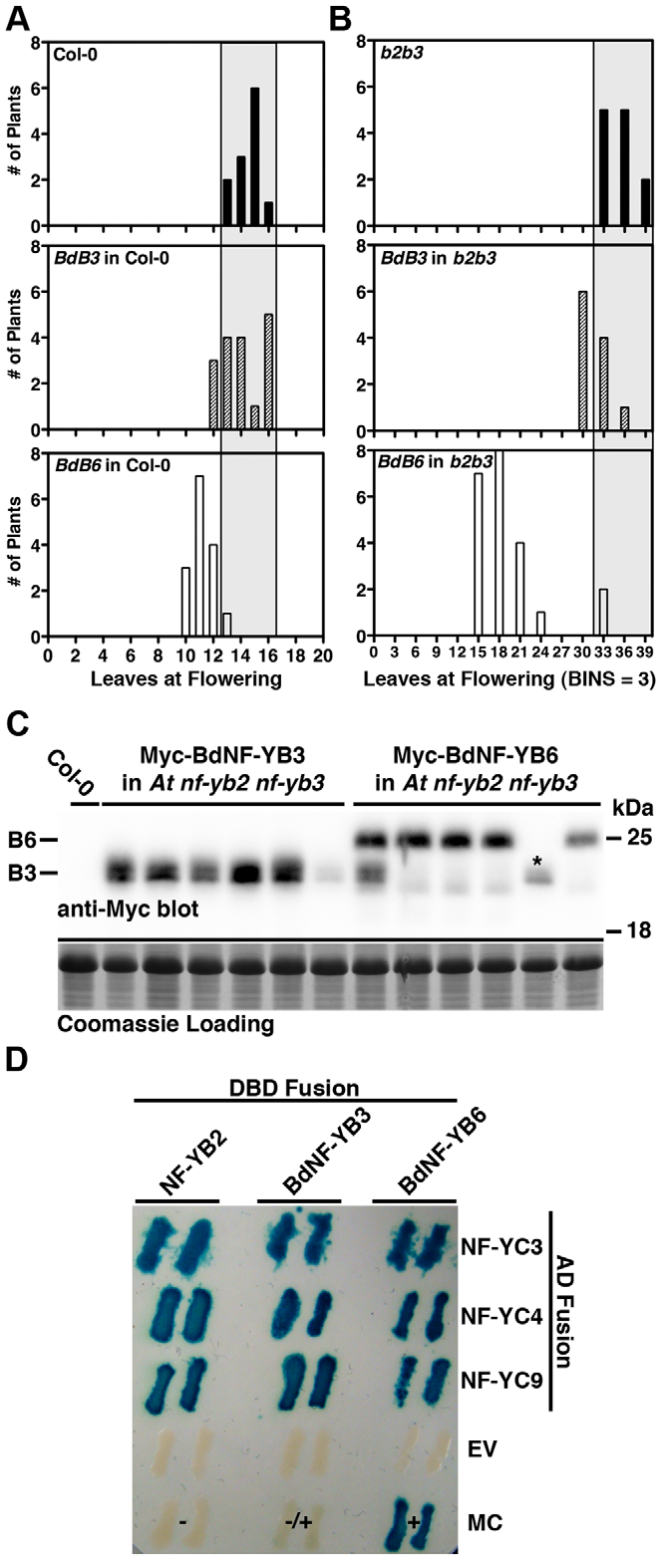
As predicted by orthology, *BdNF-YB6*, but not *BdNF-YB3*, can rescue a late flowering Arabidopsis mutant. **A**) Overexpression of *BdNF-YB6* drives early flowering in Col-0. Bars represent numbers of individual T1 plants flowering at a specific leaf number. The label in the upper left corner of each panel lists the genotype and transgene (where applicable). *BdB3* and *BdB6* are abbreviations for *35S∶BdNF-YB3* and *35S∶BdNF-YB6*, respectively. The gray bar extending from the top panel through the lower panels shows the normal range of Col-0 flowering times. Means (±SD) were 14.5 (0.90), 14.1 (1.52), and 11.2 (1.95) for Col-0, *35S∶BdNF-YB3* and *35S∶BdNF-YB6*, respectively. A non-parametric Kruskal-Wallis test (InStat 3.0, GraphPad Software) was highly significant at p<0.0001. Dunn's multiple comparison procedure (MCP) was highly significant (p<0.001) for *35S∶BdNF-YB6* compared to Col-0. **B**) Overexpression of *BdNF-YB6* in the Arabidopsis *nf-yb2 nf-yb3* mutant rescues late flowering. Means were 35.2 (1.95), 31.5 (2.02), and 19.2 (4.97) for *nf-yb2 nf-yb3*, *35S∶BdNF-YB3*, and *35S∶BdNF-YB6*, respectively. Kruskal-Wallis, p<0.0001; Dunn's MCP, p<0.001 for the *35S∶BdNF-YB6* comparison to *nf-yb2 nf-yb3*, but not different for the *35S∶BdNF-YB3* comparison to *nf-yb2 nf-yb3* or the *35S∶BdNF-YB6* comparison to Col-0. **C**) Protein blotting of independent transgenic lines (six for each transgene type) demonstrated that both BdNF-YB3 and BdNF-YB6 accumulate in the *nf-yb2 nf-yb3* background. Asterisk for BdNF-YB6 lane represents a known non-rescuing line. Coomassie staining of the transferred gel shows equal loading and equal transfer was confirmed by Ponceau red staining (not shown). **D**) Yeast two-hybrid assays demonstrate that both BdNF-YB3 and BdNF-YB6 can physically interact with Arabidopsis NF-YC3, 4, and 9. DBD, DNA-binding domain; AD, activation domain. EV - empty vector; MC - manufacturer's (Invitrogen) controls (+ strong interactor, +/− weak interactor, - non-interactor).

For each *BdNF-Y* overexpression construct and genetic background, we examined multiple independent, first generation transgenic lines. In each case, high level transgene expression was driven by the 35S cauliflower mosaic virus promoter [80]. As hypothesized, we found that *35S∶BdNF-YB6* expressed in Col-0 resulted in significantly earlier flowering in the Col-0 background (Figure 6A). *BdNF-YB6* overexpression in the *nf-yb2 nf-yb3* mutant resulted in a near full rescue of the late flowering phenotype (Figure 6B). Conversely, *BdBF-YB3* overexpression did not result in significantly earlier flowering in Col-0 or rescue of the *nf-yb2 nf-yb3* mutant phenotype (Figure 6A–B).

To confirm that these apparent functional differences were not simply due to differences in protein accumulation between the *BdNF-YB3* and *BdNF-YB6* transgenic plants, we additionally performed a protein blot analysis (Figure 6C). Six independent lines for each transgene were examined in the *nf-yb2 nf-yb3* mutant background. Bands corresponding to the expected full length proteins were readily detected for each construct. We conclude that BdNF-YB6, but not BdNF-YB3, can drive early flowering in Arabidopsis and rescue the *nf-yb2 nf-yb3* late flowering phenotype.

One simple interpretation of this data is that only BdNF-YB6 can physically interact with the appropriate Arabidopsis NF-YA and NF-YC proteins to generate floral-promoting complexes. However, this interpretation is inconsistent with previous predictions that all or most of the pairwise interactions should be possible based on the strong conservation of key amino acids in each family [41], [42]. We tested this idea by performing directed yeast two-hybrid (Y2H) interactions between BdNF-YB3 and BdNF-YB6 with the known Arabidopsis floral-promoting NF-YC proteins, NF-YC3, NF-YC4, and NF-YC9 (Figure 6D, [26]). In all instances, the Brachypodium NF-YB strongly interacted with the Arabidopsis NF-YC. Therefore, the best current explanation of the rescue data is that both BdNF-YBs can interact with the appropriate NF-YCs, but only BdNF-YB6 generates productive NF-Y complexes - i.e., can effectively bind DNA and/or recruit the appropriate trans-acting factors after dimerizing with the NF-YC. Future experiments directed towards understanding why BdNF-YB3 cannot perform some or all of these tasks will illuminate NF-Y function.

### Conclusions

NF-Y proteins are emerging as regulators of many essential and economically important developmental processes. Because most of the early NF-Y experiments have been performed in Arabidopsis, translation to the monocot lineage requires comparative analyses. We have developed a thorough initial dataset for comparisons between Arabidopsis and Brachypodium, one of the emerging monocot model systems. Helpful phylogenetic analyses have previously been performed for both wheat [16] and rice [43], and the current analyses will help bridge the gap between the dicot model system and more complex agricultural crops, especially in the case of hexaploid wheat where first demonstration experiments in a simpler system can be very useful. The demonstrated functional conservation between Arabidopsis and Brachypodium for photoperiod-dependent flowering suggests that the same principle can be extended to other process, such as BdNF-Y roles in ABA signaling and drought resistance. This additionally supports the notion of direct translation of Brachypodium results to the essential grass crops for food and biofuel production. Overall, the data presented here suggests that there is significant conservation between monocots and dicots for the three NF-Y families and future studies of these gene families and their functions in the monocot lineage will be facilitated by this comparative analysis.

## Materials and Methods

### Discovery of BdNF-Y, Multiple Alignments, and Phylogenetic Analyses

Except as detailed below, BLAST searches, multiple alignments, and phylogenetic analyses were performed as previously described [15]. To initially identify candidate BdNF-Y from each family, we compiled lists of candidates as curated at the GreenPhyl phylogenomic database [81]. We then used the BLAST [49] tool at The Arabidopsis Information Resource (TAIR, www.arabidopsis.org) to confirm that each candidate BdNF-Y from GreenPhyl best matched a known Arabidopsis NF-Y. The resulting full length sequences and conserved domains from each putative BdNF-Y was used to confirm these hits and search for additional family members at www.brachypodium.org. The conserved domains from 36 previously identified Arabidopsis NF-Y [15] were also used for BLAST searches at www.brachypodium.org. The BdNF-Y-only family trees represent full length protein sequences while the combined Brachypodium and Arabidopsis trees in supplemental data were generated from the conserved regions only.

### Plant sample preparation

The community standard diploid inbred line of *Brachypodium distachyon*, Bd21, was used for all experiments. Prior to planting, seeds were initially de-husked and sterilized by soaking in a solution of 25% household bleach (5.25% NaOCl) supplemented with 0.1% Tween-20 for 30 min with occasional rocking. Sterilized seeds were thoroughly rinsed four times with sterile water and then placed on sterile Murashige and Skoog (MS) agar (4.4 g/l MS salts with vitamins, 3% sucrose, pH 5.8, and 0.4% phytoagar). To provide sufficient vertical space for two weeks of plant growth, we used Greiner Bio-One disposable plant culture containers (VWR, Cat# 82051-508). Seeds were placed in a cold room at 4°C for four days and then transferred to a Conviron ATC13 growth chamber growth chamber at 22°C, 150 µE light intensity supplied with standard florescent bulbs. To obtain inflorescence and caryopses tissue, plants were grown in media containing equal parts Farfard C2 Mix and Metromix 200 supplemented with 40 g Marathon pesticide and dilute Peters fertilizer (NPK 20∶20∶20). After planting, seeds were stratified in a cold room at 4°C for four days prior to germination. Plants were watered with dilute Peters fertilizer at 1/10 recommended feeding levels.

### Genomic DNA extraction, total RNA isolation and primary cDNA synthesis

For each tissue type, three independent plant samples and at least five replicate samples were prepared. All tissues were flash frozen in liquid nitrogen and stored at -80°C. Prior to extraction of genomic DNA and total RNA, samples were ground in frozen mortars. DNA and RNA were extracted from Brachypodium seedlings using the Nucleon PhytoPure Kit (Amersham, RPN#8511) and E.Z.N.A. Plant RNA Kit (Omega Biotek, Cat#R6827), respectively, as per the manufacturer's instructions. Quality and quantity of extracted DNA and RNA from all samples was confirmed by both agarose gel visualization and spectrophotometry (Thermo Scientific, NanoDrop™ 1000). Prior to reverse transcription, total RNA samples were pretreated with recombinant DNase I (Ambion/Applied Biosystems, Cat#AM2235) at 37°C for 30 minutes to eliminate any contaminating genomic DNA. Primary cDNA was synthesized using the RT enzyme Superscript III (Invitrogen) per the manufacturer's instructions. Primary cDNA synthesis of all samples was performed at the same time in order to ensure approximate efficiency of reverse transcription throughout all the samples.

### Quantitative real-time PCR (qPCR) experiment using relative standard curve method

Comparisons of expression between the different BdNF-Y was performed using the relative standard curve method [82]. Genomic DNA (gDNA) was used to prepare standard curves because it has been validated as a suitable standard for mRNA expression analyses [83]. The optimal primer pair for each gene was required to have high PCR efficiency, high amplicon specificity, and approximately equal PCR efficiency between gDNA and cDNA [83]. We conducted pre-experiments to test these qualities for each primer pair and gene. The same gDNA and cDNA (a mixture of experimental cDNAs) samples were used to test all primer pairs. Calculation of PCR efficiencies were performed with the software package LinRegPCR [84], [85]. Primer pairs lacking approximately equal PCR efficiency were discarded and replaced (optimal primer pairs for each *BdNF-Y* in Table S2).

*BdNF-Y* expression analyses were then performed on cDNA samples collected from three biological replicates from each tissue type with an ABI 7500 qRT-PCR machine (Applied Biosystems, www.appliedbiosystems.com). Each 25 ul qPCR reaction included 12.5 µl 2×SYBR master mix (Fermentas, Cat#K0223), 2.5 µl primers mix (2 µM for each primer) and 5 µl gDNA or cDNA as templates. As per manufacturer recommendation, a UNG pre-treatment was performed and then the reaction was initiated by activation of Taq polymerase at 95°C for 10 min, followed by 42 cycles of 95°C for 15 s and 62°C for 1 min. Amplification specificity was determined by a final dissociation stage. Raw data were loaded into LinRegPCR and Cq (Ct) values were calculated with baseline determination and amplicon grouping. In addition to the experimental samples on each plate, a standard curve was created using a series of diluted gDNA (15 ng/µl, 3 ng/µl, 0.6 ng/µl, 0.012 ng/µl, 0.0024 ng/µl and 0.00048 ng/µl) for each primer pair. The R^2^ value for all standard curves was >0.99, except for *NF-YB4* (0.985). The standard curves were used to calibrate the experimental data. Additionally, a previously validated reference gene, UBC18 [72], was amplified in each plate and these values permitted normalization across all plates. The creation of the standard curve using gDNA, and the calibration and normalization steps followed the ABI User Bulletin and tutorials found at: http://www6.appliedbiosystems.com/support/tutorials/pdf/quant_pcr.pdf and http://www3.appliedbiosystems.com/cms/groups/mcb_support/documents/generaldocuments/cms_040980.pdf.

### DNA and transgenic plant manipulations

All target DNA fragments were generated by PCR using Pfu Ultra II (Agilent Technologies Cat#600670-51) and cloned into the GATEWAY™ entry vector pENTR/D-TOPO (Invitrogen, Cat#45-0218). Full length *BdNF-YB3* (Bradi1g43470) and *BdNF-YB6* (Bradi1g60030) were derived from Brachypodium cDNA using:

B3Forward (5′-CACCATGGAAGGACGAGGTCAAGCAAATGGC-3′)

B3Reverse (5′-TCACTCATCCACATCGTCACGGGCATG-3′);

B6Forward (5′-CAACCATGCCGGACTCTGACAATGACTCCG-3′)

B6Reverse (5′-TCAGACCCTATCTTGCCGTCCGAGGCC-3′).

All constructs were sequenced and found to be identical to the expected sequences found in the Brachypodium resource page (www.brachypodium.org). Plant rescue constructs were created using the GATEWAY™ LR Clonase II reaction kit (Invitrogen, cat#56485). Plant expression complementation constructs were subcloned from pENTR/D-TOPO into pEarlyGate203 (ABRC, Stock#CD3-689). All transgenic plants were created in the *Arabidopsis* Col-0 ecotype and transformed by the *Agrobacterium*-mediated floral dip method, as previously described [86], [87]. Arabidopsis *nf-yb2 nf-yb3* double mutants were newly generated here by standard crossing techniques from publicly available T-DNA insertion lines (SALK_025666 [23], [24] and SALK_150879, respectively). Semi-quantitative RT-PCR of mRNA from SALK_150879 showed that it is a strong knockdown line for *NF-YB3* (data not shown). For flowering time rescue experiments, Arabidopsis plants were grown under standard long day conditions (16 hr light/8 dark) as previously described [26]. Leaf number at flowering was determined by counting all primary cauline and rosette leaves immediately after the first flower opened, as previously described [88].

### Western Blot

Total protein was extracted and visualized from 28 day-old T1 plants as previously described [26], except that the following lysis buffer was used (20 mM Tris, pH 8.0, 150 mM NaCl, 1 mM EDTA, pH 8.0, 1% Triton X-100, .1% SDS; add fresh 5 mM DTT and 1X Sigma Protease Inhibitor Cocktail; cat #P9599; www.sigmaaldrich.com). Antibodies used were anti-cMyc 9E10 (primary) and goat anti-mouse (secondary, Santa Cruz Biotechnology, Cat#SC-40 and SC-2060, respectively). Protein blots were visualized on the Bio-Rad ChemiDoc XRS imaging system (www.bio-rad.com) using the horseradish peroxidase-based ECL Plus reagent (GE Healthcare, Cat#RPN2132).

### Yeast two-hybrid analysis

Gateway™ entry clones containing the full length coding regions of NF-YB2, NF-YC3, NF-YC4, NF-YC9, BdNF-YB3 and BdNF-YB6 were recombined into ProQuest™ Two-Hybrid System vectors pDEST22 and pDEST32 (Invitrogen, Cat#PQ10001-01). All interactions were tested according to instructions in the ProQuest™ manual. For the X-Gal assay nitrocellulose membranes containing yeast colonies were frozen in liquid nitrogen and incubated in Z-buffer containing X-Gal (5-Bromo-4-chloro-3-indoxyl-beta-D-galactopyranoside, Gold Biotechnology, cat#X4281L) as described in the ProQuest™ manual.

.

## Supporting Information

Figure S1 **Full-length multiple alignment for the BdNF-YA family.** Constructed using ClustalX as implemented in Mega 4.0 and previously described [91].
(PDF)

Figure S2 **Full-length multiple alignment for the BdNF-YB family.** Constructed using ClustalX as implemented in Mega 4.0 and previously described [91].
(PDF)

Figure S3 **Full-length multiple alignment for the BdNF-YC family.** Constructed using ClustalX as implemented in Mega 4.0 and previously described [91].
(PDF)

Figure S4 **Arabidopsis and Brachypodium NF-YA phylogenetic tree.** Neighbor-joining tree generated from conserved regions of Arabidopsis and Brachypodium NF-YA proteins (Datasets S1, S2) using MEGA software, version 4.0 [52]. Known functions for Arabidopsis NF-YA, NF-YB (Figure S5), and NF-YC (Figure S6) are shown in red. Phenotypes come from loss of function, overexpression (oex), and general inference (e.g., misregulation of molecular markers associated with the particular phenotype) [17], [18], [19], [20], [21], [22], [23], [24], [25], [26], [27], [28], [29], [81], [92]. No attempt was made in Figures S4, S5, S6 to distinguish between phenotypes that are strongly supported (e.g., clear loss of function phenotypes) and less well supported (e.g., inference).
(TIF)

Figure S5 **Arabidopsis and Brachypodium NF-YB phylogenetic tree.** NF-YB neighbor-joining tree constructed as in Figure S4.
(TIF)

Figure S6 **Arabidopsis and Brachypodium NF-YC phylogenetic tree.** NF-YC neighbor-joining tree is constructed as in Figure S4.
(TIF)

Table S1 **Orthology predictions between the NF-Y families from Brachypodium, Arabidopsis, rice, and wheat.** Note that the Excel file has three tabs, each corresponding to one family. Methodology notes are integrated in the table file.
(XLS)

Table S2 **Primers used for qRT-PCR expression analyses of** ***BdNF-Y*** **.**
(DOC)

Dataset S1 **Fasta file of all Brachypodium NF-Y protein sequences**. Both full length and conserved regions are included for each protein. All sequences were obtained from www.brachypodium.org.
(TXT)

Dataset S2 **Fasta file of all Arabidopsis NF-Y protein conserved regions.** Sequences were originally described in Siefers et al. [15].
(TXT)
