# Supplementary material for: Identification and Characterization of NF-Y Transcription Factor Families in the Monocot Model Plant Brachypodium distachyon
Source: PLoS One. 2011 Jun 30;6(6):e21805. doi: 10.1371/journal.pone.0021805 (PMC3128097; doi:10.1371/journal.pone.0021805)
Supplement: Figure S1 — Full-length multiple alignment for the BdNF-YA family. Constructed using ClustalX as implemented in Mega 4.0 and previously described [91]. (PDF) [file pone.0021805.s001.pdf]

```
BdNF-YA2    --------------------------------------------------------------------------MMSFKS
BdNF-YA6    --------------------------------------------------------------------------MMSFKN
BdNF-YA1    --------------------------------------------------------------------------MSGMGS
BdNF-YA7    --------------------------------------------------------------------------------
BdNF-YA3    --------------------------------------------------------------------------------
BdNF-YA4    --------------------------------------------------------------------------------
BdNF-YA5    --------------------------------------------------------------------------------
NF-YAMouse  MEQYTTNSNSSTEQIVVQAGQIQQQQGGVTAVQLQTEAQVASASGQQVQTLQVVQGQPLMVQVSGGQLITSTGQPIMVQA

BdNF-YA2    -PDGFG-QVAAVTQGSHGGGAPVP-WWAVSQLLYGEPA-ALSSP----EAE-AHRNGQFQGVPRAQGILDPAPAPPAKTA
BdNF-YA6    -YEGFGSQLAAAAASQAAGGAPLPPWWAPPQLLYGEPAGQVRSPAMFMSPEGACRDGQFQVVPRAQSLLD-AP-PPQQ--
BdNF-YA1    RPEGTN-LVEPRGQGALPSGMAMQPWWTGSGLGAVSPA--VVAPGSGIGMS-LSSNPVGDGATKGKTSDD-ARADSSE--
BdNF-YA7    ------------------------------------------------------MTSVADGIS-GDHRAD----EQQQ--
BdNF-YA3    -------MLALLRKMEDHPGHPISNYDFLSGNGCHTKKLGHKIYDQD-SSSTKSGRSQQEASAMSDSNLN-----EQHTS
BdNF-YA4    -----------MRRKEDM--H---------ENGTIMIQFGHQVPNYD-SSASDS---PQEVSGMSEGSHN-----EQN--
BdNF-YA5    ----------------------------------------MLLPASS-SSLYAS-----KGDSFGKTGDD-----DMG--
NF-YAMouse  VPGGQGQTIMQVPVSGTQGLQQIQ--LVPPGQIQIQGGQAVQVQGQQGQTQQIIIQQPQTAVTAGQTQTQQQIAVQGQ--

BdNF-YA2    QPPAKSGAPEVLKFSVFQGNLESGGKGDKTQEHSTTIALQSPLPEYNSRF-EFGPGQSMVSSNYPGADQCY-GLLTTYTM
BdNF-YA6    QTMTERGVSEGLKFSMAHG---RGGKGS---EHSTTIALQSPFSEYNDRF-ELGLGQTVLSSNYPYTDQQH-GILSHYGM
BdNF-YA1    -DSQRSGEPKDRSF----------GEEK----HHATSRMPALASDYLAPYSQLELNQPIASATYPYPDAYYTGMVGPYGA
BdNF-YA7    -QQTQAGHEDQQEA----------PATS----IGSQTMVVAPSTDYVMPYAHQEVCHAMGQIAYPSIDPYF---YGAYGG
BdNF-YA3    RSSAQSDNDDGHGKY---------NQVM----MKSPLSLGNPEAGSSPAK--VDYSQSFACM-PYTADAYYGCVLTGYAS
BdNF-YA4    ---EQSGRRDGYTNS---------DEGK----MMSALSLGNSETTYAQPK--PDRTHPFAVAYPY-ADPFYGCAYGSHAV
BdNF-YA5    -----STLN--FNNK---------PPVF----ASQNIDYGHPITRISYPY--SDSNPGLWAA--YGSRAMFHTQIAGGGT
NF-YAMouse  -QVAQTAEGQTIVYQPVNADGTILQQVTVPVSGMITIPAASLAGAQIVQTGANTNTTSSGQGTVTVTLPVAGNVVNSGGM

BdNF-YA2    KSM-----PG---GRVLLP------LNAPADAPIYVNAKQYEGILRRRRARAKVERENQLVKGRKPYLHESRHRHAMRRA
BdNF-YA6    RST-----PN---GRMLIP------LNMPADAPIYVNAKQYEAILRRRRARAKAEKENRLVKARKPYLHESRHLHAMRRA
BdNF-YA1    QAVTHFQLPGLTQSRMPLP------LEI-SEEPVYVNAKQYHGILRRRQSRAKAELERKAIKARKPYLHESRHQHAMRRA
BdNF-YA7    QPMMHPPLVGMHPAGLPLP------TDA-IEEPVYVNAKQYNAILRRRQSRAKAESERKLIKGRKPYLHESRHQHALKRA
BdNF-YA3    -H-----------AIVPVS------AESTAEEPVFVNAKQYHAILRRRQIRAKLEAQNKLVKGRKPYLHESRHRHAMKRA
BdNF-YA4    MHPQ-------IVGMVPSSRVPLPIEQAAAEEPIYVNAKQYHAILRRRQLRAKLEAENKLVKSRKPYLHESRHLHAMKRA
BdNF-YA5    STN----------TRVPLP------LELAEDEPIFVNPKQYHGILRRRQLRAKLEAQNKLIKNKKPYLHESRHRHAMKRA
NF-YAMouse  VMMVPGAGSVPAIQRIPLPG-----AEMLEEEPLYVNAKQYHRILKRRQARAKLEAEGKIPKERRKYLHESRHRHAMARK

BdNF-YA2    RGSGGRFLNTKKEGNGKAALGGGGSKRTDCTPPARLATSPNSVIPQSQLGNPHSRSSISSLSGSEVSSMYDHEDVDHYNS
BdNF-YA6    RGSGGRFLNTKKDINGKDA--GEGDKTLDSNPLMRLAASPSSEIQHSEQGN---RSSISSLSGSEVTSLYEHEDVEHYNG
BdNF-YA1    RGTGGRFLNTKKNENGASKERAEPNKGDQNSEYHRVP--PDLQLRQA---------------------------------
BdNF-YA7    RGAGGRFLNAKSDDNEEHS--DSSSKDKQNGVAPRSSGQPSTAPSSK-------GASPANQTGNRE--------------
BdNF-YA3    RGSGGRFLNTK---QLQEQKQIQASTGGKNTFLQSSPTFAPSASAPS-------DMSNVSTSSRMLANHQE--RACFPSA
BdNF-YA4    RGTGGRFLNSK---QQPE-----GSSG-------SD------ASTRA-------GHSGIPANGGMFSKHD----------
BdNF-YA5    RGSGGRFLNSKELQQQQQQQSCTVSTNATADGANSSGSTHLRLGSGS-------AGDRTTLSSKTVASQENSKRVAAPAP
NF-YAMouse  RGEGGRFFSPKEKDSPHMQDPNQADEEAMTQIIRVS--------------------------------------------

BdNF-YA2    IEHLRTPFFTPLPIIMDGEHGGAAPFKWATAADGCCELLKA
BdNF-YA6    FEQLRTHFFTPLPSIMDGEHGAGNPFRWAAASDGCCDLLRA
BdNF-YA1    -----------------------------------------
BdNF-YA7    -----------------------------------------
BdNF-YA3    GFHPMASLGAR----SGGDAELAVNGMQQRASMMIS-----
BdNF-YA4    --H---TL-------SSGDLQYRVRG---GA----------
BdNF-YA5    AFTMTPMLRKDDAFFQQHGHHLSFSGHFGQASGRYT-----
NF-YAMouse  -----------------------------------------
```
