# Supplementary material for: Identification and Characterization of NF-Y Transcription Factor Families in the Monocot Model Plant Brachypodium distachyon
Source: PLoS One. 2011 Jun 30;6(6):e21805. doi: 10.1371/journal.pone.0021805 (PMC3128097; doi:10.1371/journal.pone.0021805)
Supplement: Figure S2 — Full-length multiple alignment for the BdNF-YB family. Constructed using ClustalX as implemented in Mega 4.0 and previously described [91]. (PDF) [file pone.0021805.s002.pdf]

```
BdNF-YB1     -----------------MPDSDNE------DSG-------GGGGIGGG-GNNKEQDR--FLPIANVSRIMKKALP--ANA
BdNF-YB6     -----------------MPDSDN-------DSG-------GPSNTGGELSSPREQDR--FLPIANVSRIMKKALP--ANA
BdNF-YB12    --MKRKSYGQQQLSPEGSPPSDN-------ESGLAATMAAGGIGIGGD-SPAKEQDR--FLPIANVSRIMKRSLP--ANA
BdNF-YB8     -----------------MADAPASPGGGGGGGGSH---DSGGSPRGGGYGGVREQDR--FLPIANISRIMKKAIP--ANG
BdNF-YB9     -----------------MAD-----------GGSH---DSG-SPRGGGGGGVREQDR--FLPIANISRIMKKAVP--ANG
BdNF-YB7     -----------------MSDA----------VGTP---EEG----GGG-AGAREQER--FLPIANIGRIMRRGVP--ENG
BdNF-YB2     -MENNGEVPNTGAPSLSMDNNGGAPN-----TGAAE---AASAEEVLPVVEVREQDR--LMPIANVTRIMRRVLP--PHA
BdNF-YB4     -----------------MENSGGAPN-----TGAP--------EEVPPVVEVREQDR--LMPIANVTRIMRRMLP--PHA
BdNF-YB17    --------MEMGFAAGTAANGGGGA------AAATN---GGGGGKAPAAAAIREQDR--LMPIANVIRIMRRVLP--PHA
BdNF-YB11    -------------MADYHGGGGGSPER---RLLEAG---GGSGEQQDQGMGIKEQDR--LLPIANVGRIMKQILP--PNA
BdNF-YB10    --------MSDTFNLSGFTLPGPTPR----ISHAST---SAVGAGAGPGEEEEHQGGGGLLPIANVGRIMKGALP--PEA
NF-YBMOUSE   MTMDGDSSTTDASQLGISADYIGGSHYVIQPHDDTEDSMNDHEDTNGSKESFREQDI--YLPIANVARIMKNAIP--QTG
BdNF-YB14    -----------------MADAP---------ASP---------ASAG--GG--EEDGGFNLPIANITRIMRRAIP--PNG
BdNF-YB15    -----------------MEDAPA--------ASPRF-------GGGG--GGSREQQGGQLLPIADIGRIMRKAIP--PDG
BdNF-YB13    -----------------MADAPPP-------LCPGC-------GGRSTVGGARERDG-LLLPTANISRIMRKAIP--PGG
BdNF-YB3     -----------------MEGRGQ--------ANGP---------GNNAREGN-RRTK---FPASYLTGIMARALSQHPGA
BdNF-YB5     -----------------MEGRNQ--------ANDH---------GNNAREGNSTRTT---FPTSNLK-------------
BdNF-YB16    ------------------------------------------MDPMDIVGKSKEDVS---LPKSTMFKIIKEMLP--PDV

BdNF-YB1     KISKDAKETVQECVSEFISFITGEASDKCQREKRKTINGDDLLWAMTTLGFEDYMEPLKLYLHKFRELE----GEKAAVV
BdNF-YB6     KISKDAKETVQECVSEFISFITGEASDKCQREKRKTINGDDLLWAMTTLGFEDYVDPLKHYLHKFREIE----GERAA--
BdNF-YB12    KISKEAKETVQECVSEFISFVTGEASDKCQREKRKTINGDDLLWAMTTLGFEAYVAPLKAYLGRYREAE----GEKAAS-
BdNF-YB8     KIAKDAKETVQECVSEFISFITSEASDKCQREKRKTINGDDLLWAMATLGFEDYIEPLKVYLQKYREME----GDSKL--
BdNF-YB9     KIAKDAKETLQECVSEFISFVTSEASDKCQKEKRKTINGDDLLWAMATLGFEEYVEPLKIYLHKYRDME----GDSKL--
BdNF-YB7     KIAKDAKESIQECVSEFISFITSEASDKCMKEKRKTINGDDLIWSMGTLGFEDYVEPLKLYLKLYREME----GDTTK--
BdNF-YB2     KISDNAKELIQESTSEFISFLTGEANERCLKNRRKILTAEDILWAMDNLGFDDYVQPFTAYLQRMRDIE---NNNGVCV-
BdNF-YB4     KISDNAKELIQESTSEFISFLTGEANERCLKSRRKILTAEDILWAMDNLGFDDYVQPFTAYLQRMRDIE---NNNGVGV-
BdNF-YB17    KISDDAKETIQECVSEYISFITGEANERCQREQRKTITAEDVLWAMSRLGFDDYVHPLGVYLHRFREFEGEARGGGLGAG
BdNF-YB11    KISKEAKETMQECVSEFISFVTGEASDKCHKEKRKTVNGDDVCWAFSALGFDDYVDPMRRYLLKFRELE----GDRAAA-
BdNF-YB10    KVSKRAKEAIQECATEFVAFVTGEASQRCRRERRKTVNGDDVCHAMRSLGLDHYAAAMGRYLQRHREAE-ELAAEINGRS
NF-YBMOUSE   KIAKDAKECVQECVSEFISFITSEASERCHQEKRKTINGEDILFAMSTLGFDSYVEPLKLYLQKFREAMK---GEKGIG-
BdNF-YB14    KIDREAAEAVQELATEFIAYITLVASDICKRENQETMTGEDLLCAMYAIRLDDYMDPLNLYLDKYMSTDTGDSTEQPMDE
BdNF-YB15    DIGKDAEEAVQASVSE--------ANGKCREGKQEAVTGDHLLSAMASLGFRDYIEPLQLYLHKYR--ETGVAMDQPSEE
BdNF-YB13    KIDEDAKEAVQA------------ASDKCRREKQEAMTGHDLLWAMATLGFQDYMNPSS---------STCTSTDRLYGR
BdNF-YB3     RISGGASDAVDRCVEEFIAVVTSAAADWCREQNKTTLDSDDLINAMEDLGFYQYVGPLEDYLRQYREFQ------GRQP-
BdNF-YB5     -------DAVEQSVE-----LSPPA-----EPKKMTLEGDSVLDDMEELSFDDYVQPLSDYLRLYYELHDLVRRDDRQP-
BdNF-YB16    RVARDTQDLLVECCVEFINLLSSESNDVCSREEKKTIAPEHVIRALQDLGFKEYIEEVYAAYEQHKLDTLDSPKASKFTG

BdNF-YB1     VSGSGAGGGASQQREAPRGLGSS-------NGDGGYGGMYGG-AAAAGGGGMFMMMGQPMYG---SPP---------GYQ
BdNF-YB6     AS-TGT-------PEMPRNANAA-------AGYAGYGGASPG-TGGPAAGGM-MMM---MYG---SPP---------P-P
BdNF-YB12    VQGGGCAS--RHGGGGDDDANSSL------VSVGGSSGVLQNQNQGQEGGDVGLMMGFGAVG---TAA---------MYY
BdNF-YB8     ---------------TSKSGDGS--------VKKDTLGPHTG-TSSSSAQGMGQQV---AYN---QGM---------GYM
BdNF-YB9     ---------------TSKSGDGS--------VKKDTIGAHGG-ASSSNAQAMVQHG---AYP---QGM---------GYM
BdNF-YB7     ---------------GSRS-EQA--------GKKGIV--LNG-QPGSSFNGM----------------------------
BdNF-YB2     -------------VNNPLAAAAG-------PRAPAPVLPLVPVSVAAAAQGLMQMQRGAMYA-P-RPP---A---PVQQ-
BdNF-YB4     -------------VNNRLAAATG-------PRAPAPAPPLVPVPVAAAAQGLMQMQRGAMYA-P-RPP---A---PVQQ-
BdNF-YB17    -------------AGGSLRSPRG-------GPAPGSASSMVP---GAQHHHDMQMHAAAMYGNPMAPPHHHAFLMPQPHY
BdNF-YB11    -------------AASSRGAAAG-------FQA-GADHPAASSSAAAAAAGHFMFD--AMD----RP-------------
BdNF-YB10    -------------VGGGGVPDFG-------GQIDVRAQLSVSGSRAGAGGSEKRLGRN----------------------
NF-YBMOUSE   ---------------GAVSATDG-------LSEELTEEAFTNQLPAGLITADGQQQNVMVYT---TSY------------
BdNF-YB14    G------------MSMEQCEQPEVVLPPLHSTVVGIAEMGQEQGKDQLPDGNLKPSEIIVQQNCPEFKTLPLSSTEVTLS
BdNF-YB15    G------------MRMEQHDQSE----------EGMI-MEHEDGKDQLPNGNLRPSDIIVHKKCPEMKTLPLPS---TM-
BdNF-YB13    -------------ISIFLAIRSTS---------VGLLCIKSSR-KS--PN------------------------------
BdNF-YB3     ---------------APPTPAAL-------VTTVEMPPPQ--VPSGAAAASTSRMPPASAAAPAHARDDVDE--------
BdNF-YB5     ---------------APPPPAAA-------VTTVEMPPPPPPQPSGAAAASTSRMPPASAAAP--ARD--DE--------
BdNF-YB16    VEMS---------EEEAVAEQQRMFAEARARMNNGAAKPKESELEPQKQSQLQLHPPAHQPSQVYAQPQQPLHAQVQLHP

BdNF-YB1     QQQQQQ-QMMMGGKGGGYGSGYGDAGNGGGSSSSSGFGRQDR-M------------------------------------
BdNF-YB6     QQQQQH-QMT---RAG-FGH-QGSTG-AGGSSSSSGLGRQDR-V------------------------------------
BdNF-YB12    GAAGNN-GRR--AYGG-GGEGFQRFGGGDQEENSAGGGHLHHGVQW----------------------------------
BdNF-YB8     QPQYHN-GDISN--------------------------------------------------------------------
BdNF-YB9     QPQYHN-GDT----------------------------------------------------------------------
BdNF-YB7     --------------------------------------------------------------------------------
BdNF-YB2     -QGYAI-GAMPLQVRAPAPVVGGQQGSVLGGKRPVGDGEGSSRGD-VGD-EAS---------------------------
BdNF-YB4     -QGYAI-GAMPLQVRAPPPPLGGQ--------RPVGDGEGSSRGEKPADGEGSSR-------------------------
BdNF-YB17    GQQYEMYGGGEHGMGAAAYYGGGYAPGNGGHNGDSGSGGGGANTPQAVNFEHQQPFGYK---------------------
BdNF-YB11    -DNNNS-GSRQF--------------------------------------------------------------------
BdNF-YB10    --------------------------------------------------------------------------------
NF-YBMOUSE   -QQISGVQQIQFS-------------------------------------------------------------------
BdNF-YB14    EVLLCPMEVEPIALHVPDIRHWSELPLDALSAIFMKLGTIEILMGAGLVCRPWLAAAKSPELWRFVDMTRHKVVFSKSEN
BdNF-YB15    -VLPRTMEAEPKPLPVPEVRDWSELTVDALSAIFTKLGTIEILMGAGLVCHSWLEAAKLPDLWRFVSCPRHNVVFSKAGD
BdNF-YB13    --------------------------------------------------------------------------------
BdNF-YB3     --------------------------------------------------------------------------------
BdNF-YB5     --------------------------------------------------------------------------------
BdNF-YB16    QPQQPLHGQVQLHPQAQSQQPLHAQVQLHPQAQPQQPLHAQVQLHPQPQQPLQPQLQLQPQAQPPQTFHPQPQLQLQPQA
```

```
BdNF-YB1     --------------------------------------------------------------------------------
BdNF-YB6     --------------------------------------------------------------------------------
BdNF-YB12    --------------------------------------------------------------------------------
BdNF-YB8     --------------------------------------------------------------------------------
BdNF-YB9     --------------------------------------------------------------------------------
BdNF-YB7     --------------------------------------------------------------------------------
BdNF-YB2     --------------------------------------------------------------------------------
BdNF-YB4     --------------------------------------------------------------------------------
BdNF-YB17    --------------------------------------------------------------------------------
BdNF-YB11    --------------------------------------------------------------------------------
BdNF-YB10    --------------------------------------------------------------------------------
NF-YBMOUSE   --------------------------------------------------------------------------------
BdNF-YB14    IMLKMAKVAIDRSDGRMESFWAQKFVSGELLDYIASRGNSLKSIRLIACGFCWDGAVTRLAAKCQMLEEIEYSHQKQPGD
BdNF-YB15    VMCKMAKVAVDRSDGRMESFWAQKFVSSELLDYIASRSNDAR-----------DDHGTVL---CN-----------DPRD
BdNF-YB13    --------------------------------------------------------------------------------
BdNF-YB3     --------------------------------------------------------------------------------
BdNF-YB5     --------------------------------------------------------------------------------
BdNF-YB16    EPPQTVQPQLQFHPQPQQTIPPLLQPQPQQPPPQPELQLHHQSEQPTQAQLQPQAQEPQLQLQPQSQTEHGLGSS-----

BdNF-YB1     --------------------------------------------------------------------------------
BdNF-YB6     --------------------------------------------------------------------------------
BdNF-YB12    --------------------------------------------------------------------------------
BdNF-YB8     --------------------------------------------------------------------------------
BdNF-YB9     --------------------------------------------------------------------------------
BdNF-YB7     --------------------------------------------------------------------------------
BdNF-YB2     --------------------------------------------------------------------------------
BdNF-YB4     --------------------------------------------------------------------------------
BdNF-YB17    --------------------------------------------------------------------------------
BdNF-YB11    --------------------------------------------------------------------------------
BdNF-YB10    --------------------------------------------------------------------------------
NF-YBMOUSE   --------------------------------------------------------------------------------
BdNF-YB14    FFKQLGAVRPELKRLRIHMQWFDSDAIEREMREEQQSSHDEDEEEEEEEEEPYEAWEMRHNEEAFAIAENLHELRLLQMA
BdNF-YB15    --EHGTAQSPE--------QYHDED-------DDNGDTAADDEVGPFFPGFPYGDWEARHNRVAFAIAANLHELQLLQLA
BdNF-YB13    --------------------------------------------------------------------------------
BdNF-YB3     --------------------------------------------------------------------------------
BdNF-YB5     --------------------------------------------------------------------------------
BdNF-YB16    --------------------------------------------------------------------------------

BdNF-YB1     --------------------------------------------------------------------------------
BdNF-YB6     --------------------------------------------------------------------------------
BdNF-YB12    --------------------------------------------------------------------------------
BdNF-YB8     --------------------------------------------------------------------------------
BdNF-YB9     --------------------------------------------------------------------------------
BdNF-YB7     --------------------------------------------------------------------------------
BdNF-YB2     --------------------------------------------------------------------------------
BdNF-YB4     --------------------------------------------------------------------------------
BdNF-YB17    --------------------------------------------------------------------------------
BdNF-YB11    --------------------------------------------------------------------------------
BdNF-YB10    --------------------------------------------------------------------------------
NF-YBMOUSE   --------------------------------------------------------------------------------
BdNF-YB14    GNSLTKKGVYAILEGCPHLECLDLTECDHLKVDDELLARCAKIRHVWLPGRWPRVHCPDLHTIGEDEGEVIEMDDVYEIE
BdNF-YB15    ADSLTNGGVYTILDGCPRLECVDLTACRHLQVDDELLARCAKLTHVWLPGRWPRVHCPDLRNI------CFEQDDLREME
BdNF-YB13    --------------------------------------------------------------------------------
BdNF-YB3     --------------------------------------------------------------------------------
BdNF-YB5     --------------------------------------------------------------------------------
BdNF-YB16    --------------------------------------------------------------------------------

BdNF-YB1     --------------------------------------------------------
BdNF-YB6     --------------------------------------------------------
BdNF-YB12    --------------------------------------------------------
BdNF-YB8     --------------------------------------------------------
BdNF-YB9     --------------------------------------------------------
BdNF-YB7     --------------------------------------------------------
BdNF-YB2     --------------------------------------------------------
BdNF-YB4     --------------------------------------------------------
BdNF-YB17    --------------------------------------------------------
BdNF-YB11    --------------------------------------------------------
BdNF-YB10    --------------------------------------------------------
NF-YBMOUSE   --------------------------------------------------------
BdNF-YB14    ACALRDEGAMERGNDDYGDNYWDDYSLPSSPGSPDLPDVTCDDTRYYTYIHEYYSL
BdNF-YB15    ARVLHNEAAVEYVG-EYADNYYWEYSSGSSSQMSDLSDVAWDD-------------
BdNF-YB13    --------------------------------------------------------
BdNF-YB3     --------------------------------------------------------
BdNF-YB5     --------------------------------------------------------
BdNF-YB16    --------------------------------------------------------
```
