# Supplementary material for: Identification and Characterization of NF-Y Transcription Factor Families in the Monocot Model Plant Brachypodium distachyon
Source: PLoS One. 2011 Jun 30;6(6):e21805. doi: 10.1371/journal.pone.0021805 (PMC3128097; doi:10.1371/journal.pone.0021805)
Supplement: Figure S3 — Full-length multiple alignment for the BdNF-YC family. Constructed using ClustalX as implemented in Mega 4.0 and previously described [91]. (PDF) [file pone.0021805.s003.pdf]

```
BdNF-YC1     MAGKKRAAATPAPPPSANCPDGAVASIPSAKRGRGRPKSTAPTAAKRGPGSAAPNSGAASELAAPKPGKIGALKKKEPQE
BdNF-YC2     --------ME----P---------SSQP---Q---PAVGAVASGSEVY-AASNYS-PAAAVAV---APGVI-----PAAS
BdNF-YC5     --------ME----P---------SSQP---E---PVMGVATAGSQAYPPAAAYP-PPGMVPG---APAII-----PPGA
BdNF-YC10    --------ME----P-K-------STTP---PPPAPVLGAPVG----YPPAAVYP-PSAAAAGYPHAPALYAPPPPPPPA
BdNF-YC12    --------MD----PTK-------SSTP---PPP-PVLGAPVG----YPPGAYSPQPGAAAAAY--APQLYA----PPAA
BdNF-YC3     --------MDNHQLPYS-------AQTP-----------ATTGGGAPVPAGATGP---------------------PPAV
BdNF-YC6     --------MDQHSQTKVE-DVVMVSGAP--------------------SAGTAFP------AGSNGGPVVYAA---APLQ
BdNF-YC7     --------MNQHSQPRTEADAT--NDTPVAYVA-GTVYGVAPVG-VIFPAGTVFH------VGPRGGP-IYAA---TPVK
BdNF-YC8     --------MDGHSQPWAEADATNVNSTPVAYVAPGTVSGAAPAGAAAFPAGTVFA------AGSSGGPVVYAA---TPLQ
BdNF-YC9     --------------------------------------------------------------------------------
NF-YCMouse   --------------------------------------------------------------------------------
BdNF-YC4     --------------------------------------------------------------------------------
BdNF-YC11    --------------------------------------------------------------------------------

BdNF-YC1     EGSKRKKQQAAGGAEKATTPAKMMKKKGGGEAEPGSRKKKGKQESSGEAAEKPATAKKKKQASNGAEKATSPGKRKRGDA
BdNF-YC2     QQAPPFAGNPALLSAQNQLVYQQAQ-------------------------------------------------------
BdNF-YC5     QSTVPFPTNPAQLSAQHQLVYQQAQ-------------------------------------------------------
BdNF-YC10    AAS-----------------------------------------------------------------------------
BdNF-YC12    AAA-----------------------------------------------------------------------------
BdNF-YC3     PQ------------------------------------------------------------------------------
BdNF-YC6     Q-------------------------------------------------------------------------------
BdNF-YC7     QEDQHQQQLQAFWTDRLDEIEHMSDFKIHSLPLARIKKIMKASGENVHMIAGEAPGVLPKACEIFIQELTLRSWLQTREK
BdNF-YC8     Q-------------------------------------------------------------------------------
BdNF-YC9     --------------------------------------------------------------------------------
NF-YCMouse   --------------------------------------------------------------------------------
BdNF-YC4     --------------------------------------------------------------------------------
BdNF-YC11    --------------------------------------------------------------------------------

BdNF-YC1     EKPKSAKKAPAAAEKPTPTK---------------------------------------------------RKKKEDGEA
BdNF-YC2     --------------------------------------------------------------------QFHQQLQQHQQG
BdNF-YC5     --------------------------------------------------------------------QFHEQLQQQQQQ
BdNF-YC10    -----------------------------------------------------------------------QQAAAAQQQ
BdNF-YC12    -----------------------------------------------------------------------QQAAAA-QQ
BdNF-YC3     -----------------------------------------------------------------------HHLLQQQQA
BdNF-YC6     --------------------------------------------------------------------QLPLQQKQEVQQ
BdNF-YC7     NRRTLLRHKCMYLLAMDQHSQPRTEADATNDTSVAYLAGTESGPAPAGVVFPAGTVFHVGPYGGPVYAALPMQQEDQHQQ
BdNF-YC8     -----VRH--------------------------------------------------------------PLQQEDQHQQ
BdNF-YC9     --------------------------------------------------------------------------------
NF-YCMouse   ---------------------------------------------------------------MSTEGGFGGTSSSDAQQ
BdNF-YC4     -----------------------------------------------------------------------------MRK
BdNF-YC11    -----------------------------------------------------------------------------MRK

BdNF-YC1     EAKSGKKKGSPAKKAAAAAEPGSCSFPMSRVRLLMR-D-EDASMRATNETVFLINKASELFLEAFAKDAHQNALKERKKS
BdNF-YC2     QLQQFWAERLSEIE--QTTDFKNHTLPLARIKKIMKAD-EDVRMISA-EAPVIFAKACEIFILELTLRSWMHTEENKRRT
BdNF-YC5     QLRDFWASQMVEIE--QAADFKNHTLPLARIKKIMKAD-EDVRMISA-EAPVVFAKACEVFILELTLRSWMHTEENKRRT
BdNF-YC10    QLQMFWAEQYREIE--ATTDFKNHNLPLARIKKIMKAD-EDVRMIAA-EAPVVFARACEMFILELTHRGWAHAEENKRRT
BdNF-YC12    HLQMFWAEQYREIE--ATTDFKNHNLPLARIKKIMKAD-EDVRMIAA-EAPVVFARACEMFILELTHRGWAHAEENKRRT
BdNF-YC3     QLQAFWAYQRQEAERASASDFKNHQLPLARIKKIMKAD-EDVRMISA-EAPVLFAKACELFILELTIRSWLHAEENKRRT
BdNF-YC6     QLQTFWADRKTEIE--QITDCKTHSLPLARIKKIMKAD-EDVQMIAG-EAPAVFAKACEMFILELTLRSWLQTRENNRNT
BdNF-YC7     QLQAFWSDRLDEIE--HMSDFKTHSLPLARIKKIMKASGENVQMIAG-EAHGLLAKACEIFIQELTLRSWLQTRENNRRT
BdNF-YC8     KLQDFWTETLAEIE--HMSEIKPHSLPLARIKKIMKASGEDIRMIAS-EAPGLLAKASEIFIQELTLRSWLETRDNNRRT
BdNF-YC9     -----------------MSDFKIHSLPLERIKKIMKASGENVQVIAG-EAPGVLTKACEIFIQELTLRSWLQTREKNRRT
NF-YCMouse   SLQSFWPRVMEEIRNLTVKDFRVQELPLARIKKIMKLD-EDVKMISA-EAPVLFAKGAQIFITELTLRAWIRTEDNKRRP
BdNF-YC4     KLDT--------------------RFPAPRIKKIMQAD-EDVGKIAL-AVPVLVSKALELFLQDLCDRTYNITVQKGVKT
BdNF-YC11    KLGT--------------------RFPAARIKKIMQAD-EDVGKIAL-AVPVLVSRALELFLQDLIDRSYNITVQSGAKT

BdNF-YC1     IAYDNLSTSVCNQKRYKFLSDFVPLR------------------------------------------------VTAG--
BdNF-YC2     LQKNDIAAAITRTDIYDFLVDIIPRD----------------------EMKEEG------VGLPRAG-PLP-LGAP-A--
BdNF-YC5     LQKNDIAAAITRTDIYDFLVDIIPRD----------------------DMKEEG------LGLQRVGLPPP-PGAP-A--
BdNF-YC10    LQKSDIAAAIARTEVFDFLVDIVPRD----------------------EAKDAE------AAAVAAGMPHPAAGMPTA--
BdNF-YC12    LQKSDIAAAIARTEVFDFLVDIVPRD----------------------DAKDADAAAAAAAAAAAAGIPRPAAGVPAT--
BdNF-YC3     LQRNDVAAAIARTDVFDFLVDIVPRE----------------------EAKEEPGSSALGFAGAGAG---P-GGAP-A--
BdNF-YC6     LQKNDIATVVSRNDDFDFLVDV---------------------------MQENG------AVLPPVTLQTMVPGM--G--
BdNF-YC7     LQKNDIAAAVSRNEAFDFLVDI---------------------------MQDNG------AGLPTGTMQTMVPGM--G--
BdNF-YC8     LQKNDIGAAVSRNETFDFLVDV---------------------------MQDNG------VGFPSATVQTAVLGM--S--
BdNF-YC9     LQKNDIAAAVSRNEAFDFLVDI---------------------------MQDNG------VGLPTGTMQTMIPGM--G--
NF-YCMouse   LQRNDIAMAITKFDQFDFLIDIVPRD----------------------ELK------------PPKRQEEVRQSVTPA--
BdNF-YC4     VSSSHLKQCIHSYDVYDFLKNVVNKVPDLGAPDTSADDK-LGKRRK--HAEDESEEESKRTRNEVASHASNGRGRGRGRG
BdNF-YC11    LNSFHLKQCVKRYNSFDFLTEIVNKVPDLGGADSCGDERGLPRRRKLSNESDPENEEPRSSKMPIRSLNTSPRGRGRGRG

BdNF-YC1     ------------------DALKATAVD-----------------------------------------------------
BdNF-YC2     ------------------DPYPYYYLP--QQQVPGAAMVYAGQQGHPMTYAW----------------------------
BdNF-YC5     ------------------EAYPYYYVP--AQQVPGVGMMYGGQQGHPVTYAW----------------------------
BdNF-YC10    ------------------DSMAYYYVP--PQ-------------------------------------------------
BdNF-YC12    ------------------DPMAYYYVP--QQ-------------------------------------------------
BdNF-YC3     ------------------AGLPYYYPP--MG------------QPAPMMPAW----------------------------
BdNF-YC6     ------------------IPFGMY-GN---Q--------------LPTAFAW----------------------------
BdNF-YC7     ------------------T-FEMYCGN---Q--------------QPVPFAW----------------------------
BdNF-YC8     ------------------T-FGMYYGN--QQ--------------QPVPFAW----------------------------
BdNF-YC9     ------------------T-FGMYYENLTSNQFLSRGRSRSSSSRLTTLPSWNHNRRTLQKNDIAATVSRNDTFDFLMDI
NF-YCMouse   ------------------EPVQYYFTL---AQQPTAVQVQGQQQPQQTTSST----------------------------
BdNF-YC4     RGRRGGRGAERETEYHELAPCESKPVNLKVEIGDVGSDTIETKEPTPLSNAR----------------------------
BdNF-YC11    RGR--GR-----------PPTKRKEVGY-VQFEDESSMFTEQSESLPGDDAI----------------------------
```

```
BdNF-YC1     --------------------------------------------------------------------------------
BdNF-YC2     ----------------------------------------------------------------------------Q---
BdNF-YC5     ----------------------------------------------------------------------------Q---
BdNF-YC10    --------------------------------------------------------------------------------
BdNF-YC12    --------------------------------------------------------------------------------
BdNF-YC3     ----------------------------------------------------------------------------HVPA
BdNF-YC6     ----------------------------------------------------------------------------P---
BdNF-YC7     ----------------------------------------------------------------------------P---
BdNF-YC8     ----------------------------------------------------------------------------L---
BdNF-YC9     MQENENKPVHTEEVHLGHGSTLTATNNTLAMDQPIQALGRVHLDALGRAGDGVDEDEDNAVAPRGRPAAVSRIVGAQAKK
NF-YCMouse   ----------------------------------------------------------------------TTIQPGQIII
BdNF-YC4     --------------------------------------------------------------------------------
BdNF-YC11    --------------------------------------------------------------------------------

BdNF-YC1     -KP-----------------------------------------------------------------------------
BdNF-YC2     -DP----------------------------QEQQ----QQ-----QQGAPAEQQSLHDSG-------------------
BdNF-YC5     -QP----------------------------QGQQVETPEE-----QQQSP-----------------------------
BdNF-YC10    --------------------------------------------------------------------------------
BdNF-YC12    --------------------------------------------------------------------------------
BdNF-YC3     WDP----------------------------AWQQGGADV-----DQAAAGGGFGAEDGQGFTGHGGPAGYPSGPPSSE
BdNF-YC6     -QP----------------------------EQQ-PPYNGE-----QQQEEE-EEEEEEPPYNGGQDV------------
BdNF-YC7     -QP----------------------------QQQQSPHNGE-----QQHQPPYNGEQQQPPSSGGQDE------------
BdNF-YC8     -QP----------------------------EHQPPPYNGE-----QQQQPP-------PPSSDGQDE------------
BdNF-YC9     TQPLGKAPDSFMCGRAGAGCAEAAAELDLWRRLRHPSSSGE-----QRDASGGGRREESGIGLSPATMQTIVPAGDRHSL
NF-YCMouse   AQPQQGQTTPVTMQVGEGQQVQIVQAQPQGQAQQTQSGTGQTMQVMQQIITNTGETQQIPVQLNAAQLQYIRLAQPVSGT
BdNF-YC4     -ASLRNIDLNLDLDEYEDTVAQFQPEAPVASVAAPSAG-PSVSQSIEDVKTKDFLGWQLPE-MSKMGMDPVQFALSSDH-
BdNF-YC11    -PETK-------------CGSESIPQSANPPADAPSTGVPAAISKVEEASTNHQPDWPMPDAIGGIGVGPSSFG----HL

BdNF-YC1     ---------------------------------------------------------------------------
BdNF-YC2     ---------------------------------------------------------------------------
BdNF-YC5     ---------------------------------------------------------------------------
BdNF-YC10    ---------------------------------------------------------------------------
BdNF-YC12    ---------------------------------------------------------------------------
BdNF-YC3     ---------------------------------------------------------------------------
BdNF-YC6     ---------------------------------------------------------------------------
BdNF-YC7     ---------------------------------------------------------------------------
BdNF-YC8     ---------------------------------------------------------------------------
BdNF-YC9     SLGRSRSSSSSHLRMVDKRN-------------------------------------------------------
NF-YCMouse   QVVQGQIQTLATNAQQITQTEVQQGQEQFSQFTDGQQLYQIQQVTMPAGQDLAQPMFIQSANQPSDGQTPQVTGD
BdNF-YC4     --RLEVDEDYDNEE-------------------------------------------------------------
BdNF-YC11    TVQVDEDEDYDNED-------------------------------------------------------------
```
