# Supplementary figures and images for: Identification and Characterization of NF-Y Transcription Factor Families in the Monocot Model Plant Brachypodium distachyon
Source: PLoS One. 2011 Jun 30;6(6):e21805. doi: 10.1371/journal.pone.0021805 (PMC3128097; doi:10.1371/journal.pone.0021805)

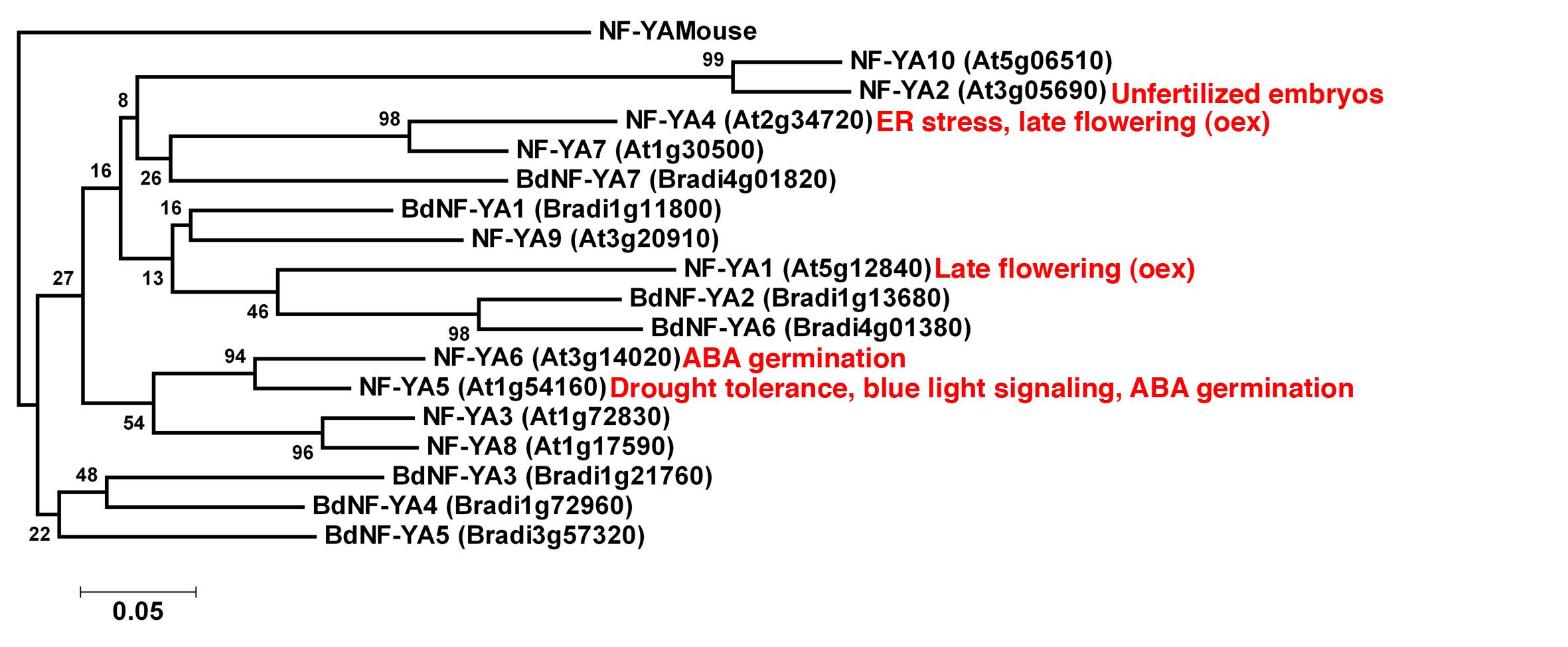

Supplement: Figure S4 — Arabidopsis and Brachypodium NF-YA phylogenetic tree. Neighbor-joining tree generated from conserved regions of Arabidopsis and Brachypodium NF-YA proteins (Datasets S1, S2) using MEGA software, version 4.0 [52]. Known functions for Arabidopsis NF-YA, NF-YB (Figure S5), and NF-YC (Figure S6) are shown in red. Phenotypes come from loss of function, overexpression (oex), and general inference (e.g., misregulation of molecular markers associated with the particular phenotype) [17], [18], [19], [20], [21], [22], [23], [24], [25], [26], [27], [28], [29], [81], [92]. No attempt was made in Figures S4, S5, S6 to distinguish between phenotypes that are strongly supported (e.g., clear loss of function phenotypes) and less well supported (e.g., inference). (TIF) [file pone.0021805.s004.tif]

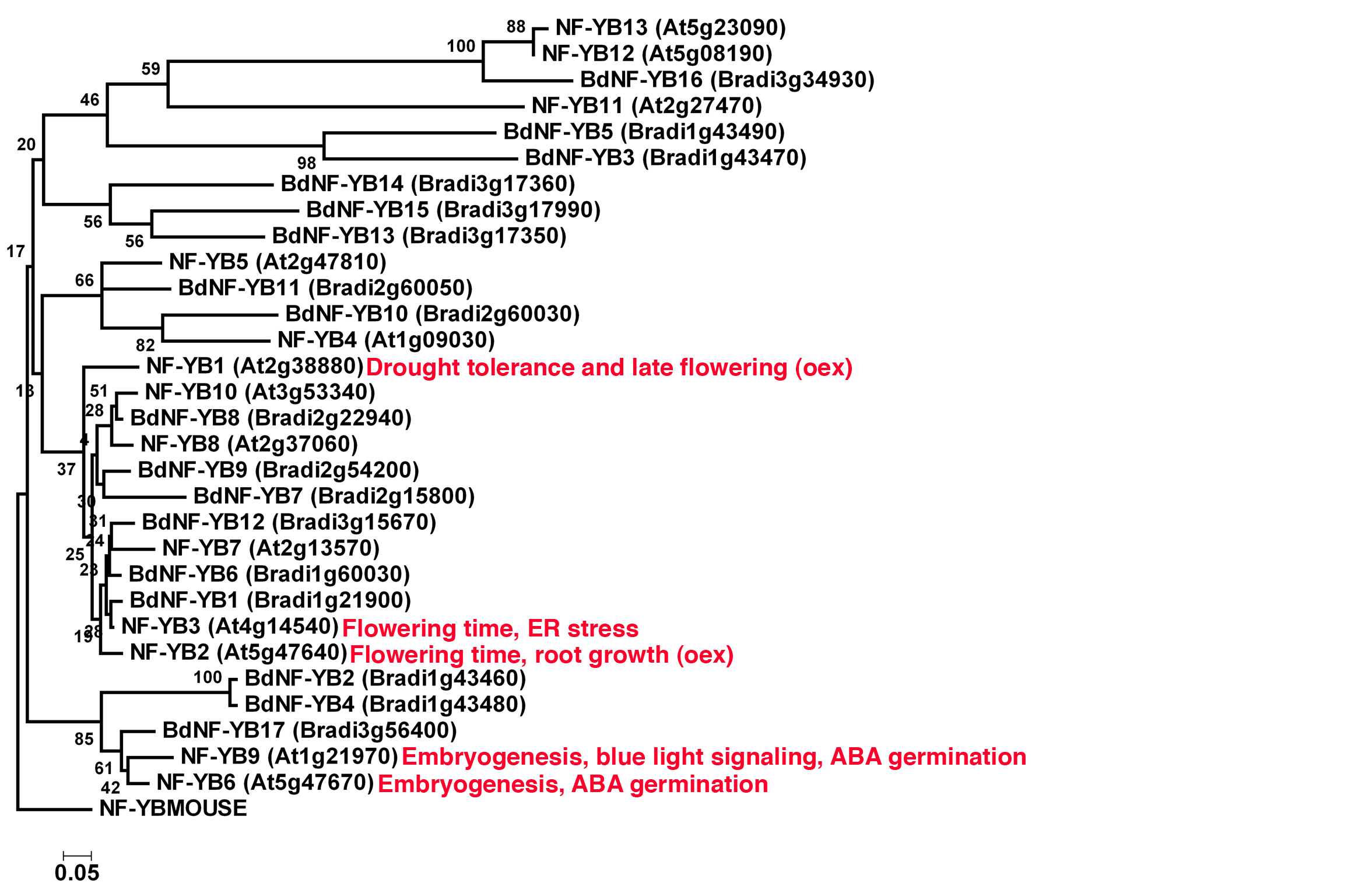

Supplement: Figure S5 — Arabidopsis and Brachypodium NF-YB phylogenetic tree. NF-YB neighbor-joining tree constructed as in Figure S4. (TIF) [file pone.0021805.s005.tif]

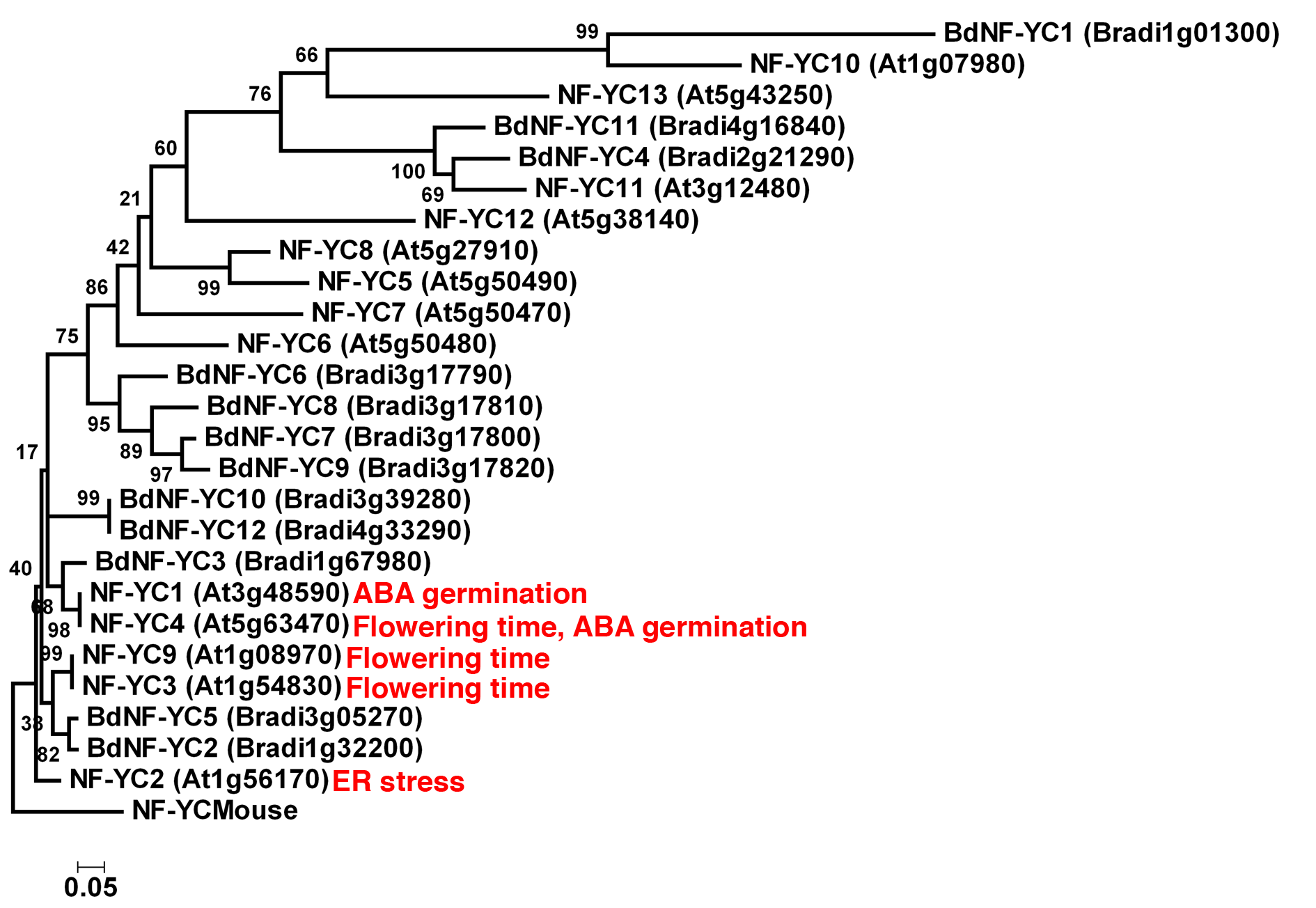

Supplement: Figure S6 — Arabidopsis and Brachypodium NF-YC phylogenetic tree. NF-YC neighbor-joining tree is constructed as in Figure S4. (TIF) [file pone.0021805.s006.tif]
