# Supplementary material for: Identification and Characterization of NF-Y Transcription Factor Families in the Monocot Model Plant Brachypodium distachyon
Source: PLoS One. 2011 Jun 30;6(6):e21805. doi: 10.1371/journal.pone.0021805 (PMC3128097; doi:10.1371/journal.pone.0021805)
Supplement: Table S2 — Primers used for qRT-PCR expression analyses of BdNF-Y . (DOC) [file pone.0021805.s008.doc]

| **Genes** | **IBI#** | **Forward primer (5'-----3')** | **Reverse primer (5'-----3')** | **Band size (bp)** |
| --- | --- | --- | --- | --- |
| **BdNF-YA1** | Bradi1g11800 | **TGCGAAGGGCAAGAGGAACTGGG** | **TGGTCGCCTTTGTTCGGTTCAGC** | 97 |
| **BdNF-YA2** | Bradi1g13680 | **TCGAGCACCTCCGGACACCCTTC** | **CATCGGCAGCTGTGGCCCACTTG** | 99 |
| **BdNF-YA3** | Bradi1g21760 | **CCTGCTTTCCATCGGCCGGCTTC** | **GCACGCTGCTGCATGCCATTAAC** | 97 |
| **BdNF-YA4** | Bradi1g72960 | **TCAAAGCAGCAGCCCGAGGGTTC** | **ATCGTGCTTCGAGAACATGCCAC** | 99 |
| **BdNF-YA5** | Bradi3g57320 | **TGCCCCCGCCTTCACCATGA** | **CCCGCTTGCCTGGCCAAAGT** | 106 |
| **BdNF-YA6** | Bradi4g01380 | **TTCACCCCGCTGCCGAGCAT** | **ATCAGAGGCTGCTGCCCACCTG** | 75 |
| **BdNF-YA7** | Bradi4g01820 | **AACAGAACGGAGTTGCGCCCCG** | **ACGATTGCCCGTCTGGTTCGCT** | 92 |
| **BdNF-YB1** | Bradi1g21900 | **ATGATGGGAGGCAAAGGCGGCG** | **CACATCCTGTCTTGCCTGCCGAAC** | 104 |
| **BdNF-YB2** | Bradi1g43460 | **GCCATCGGCGCCATGCCATT** | **AACGGGGCGCTTTCCACCCA** | 90 |
| **BdNF-YB3** | Bradi1g43470 | **ACCGGCAGCCCTTGTGACGA** | **ACATCGTCACGGGCATGGGCA** | 126 |
| **BdNF-YB4** | Bradi1g43480 | **GCAGCAGCAGGGGTATGCCATC** | **GCCTTCCCCGTCCGCAGGTTTT** | 133 |
| **BdNF-YB5** | Bradi1g43490 | **ACGACCGTCAGCCTGCACCAC** | **GCAGCAGCAGAAGCAGGTGGCA** | 133 |
| **BdNF-YB6** | Bradi1g60030 | **CGCCACAGCAGCAGCAGCAA** | **GACCCTATCTTGCCGTCCGAG** | 113 |
| **BdNF-YB7** | Bradi2g15800 | **GGAGATGGAGGGTGACACAAC** | **CGTTGAACGATGATCCAGGTTGTC** | 95 |
| **BdNF-YB8** | Bradi2g22940 | **TTCCTGCCCATCGCCAACATCAG** | **AACTCGGAGACGCACTCCTGCAC** | 107 |
| **BdNF-YB9** | Bradi2g54200 | **TTGGTGCCCATGGTGGTGCCAG** | **ACTGAGGTTGCATGTAGCCCATCCC** | 93 |
| **BdNF-YB10** | Bradi2g60030 | **CGGATTTCGGAGGCCAGATCGAC** | **TGCCAAGGCGCTTCTCGCTG** | 90 |
| **BdNF-YB11** | Bradi2g60050 | **ACCACCCTGCTGCTTCCTCTTC** | **TCAAAACTGCCTGGAGCCGCTGT** | 107 |
| **BdNF-YB12** | Bradi3g15670 | **TGTTGGGACGGCGGCGATGT** | **CCGCCGAACCGCTGAAACCCTT** | 93 |
| **BdNF-YB13** | Bradi3g17350 | **ACGAGGATGCCAAGGAGGCCGT** | **AGTCCTGAAACCCCAGCGTGGC** | 118 |
| **BdNF-YB14** | Bradi3g17360 | **TCTGCGCGACGAGGGAGCAA** | **ACACGTCACGTCGGGCAAGTCG** | 115 |
| **BdNF-YB15** | Bradi3g17990 | **ACGTGAAATGGAGGCTCGTGTCCTT** | **TGGGACGAGGAACCCGAGGAGT** | 105 |
| **BdNF-YB16** | Bradi3g34930 | **TCAATCAGAGCAGCCGACTCAAG** | **AAGCCATGTTCCGTTTGCGACTG** | 93 |
| **BdNF-YB17** | Bradi3g56400 | **GGACATAATGGAGACAGCGGCAG** | **TACTTGTACCCGAACGGCTGCTG** | 92 |
| **BdNF-YC1** | Bradi1g01300 | **ATGTCGCGGGTGCGGTTGCT** | **AGCTCCGAGGCCTTGTTGATGAGG** | 92 |
| **BdNF-YC2** | Bradi1g32200 | **CCAGGTGCAGCAATGGTGTATG** | **TGCTGCTGCTGCTGTTGTTCCTG** | 89 |
| **BdNF-YC3** | Bradi1g67980 | **TGCCGTACTACTACCCGCCCATG** | **TCCGGCAGCAGCCTGATCCA** | 122 |
| **BdNF-YC4** | Bradi2g21290 | **AGACCAAGGATTTCCTGGGCTG** | **CATAATCTATGGTCCGACGACAG** | 94 |
| **BdNF-YC5** | Bradi3g05270 | **TGGGGCACCAGCTGAAGCATATC** | **TGCTGCTGCTCTTCAGGGGTCTC** | 156 |
| **BdNF-YC6** | Bradi3g17790 | **CGCGGTGTTGCCACCTGTGA** | **GCTGCGGCCAAGCGAAAGCA** | 104 |
| **BdNF-YC7** | Bradi3g17800 | **AGTTCCTTTCGCGTGGCCGCA** | **TGGAAGGCGGCTGTTGCTGCT** | 104 |
| **BdNF-YC8** | Bradi3g17810 | **GGTCCTGGGAATGAGCACCTTTG** | **GCTGCAGCCACGCGAAAGGA** | 74 |
| **BdNF-YC9** | Bradi3g17820 | **GGCGGCGCGAGGAAAGTGGTAT** | **TCCGGCTGCGGCCAAGTGAA** | 99 |
| **BdNF-YC10** | Bradi3g39280 | **TCCTTGTTGACATCGTGCCGAG** | **CGTAGTAGTAGGCCATGGAGTC** | 122 |
| **BdNF-YC11** | Bradi4g16840 | **GGGTGCCAGCTGCAATTTCAAAG** | **ACCAATGCCTCCAATGGCGTCTG** | 90 |
| **BdNF-YC12** | Bradi4g33290 | **TAGACATCGTGCCACGTGATGAC** | **ACATAGTAGTAAGCCATCGGGTC** | 133 |

**Table S2. Primers used to amplify Brachypodium NF-Y for qRT-PCR analyses.**
